# Automating quantum computing laboratory experiments with an agent-based AI framework

**DOI:** 10.1016/j.patter.2025.101372

**Published:** 2025-09-23

**Authors:** Shuxiang Cao, Zijian Zhang, Mohammed Alghadeer, Simone D. Fasciati, Michele Piscitelli, Mustafa Bakr, Peter Leek, Alán Aspuru-Guzik

**Affiliations:** 1 Clarendon Laboratory, Department of Physics, University of Oxford, Oxford OX1 3PU, UK; 2 Department of Computer Science, University of Toronto, Toronto, ON M5S 2E4, Canada; 3 Vector Institute for Artificial Intelligence, Toronto, ON M5G 1M1, Canada; 4 Department of Chemistry, University of Toronto, Toronto, ON M5S 3H6, Canada; 5 Department of Materials Science & Engineering, University of Toronto, Toronto, ON M5S 3E4, Canada; 6 Department of Chemical Engineering & Applied Chemistry, University of Toronto, Toronto, ON M5S 3E5, Canada; 7 Canadian Institute for Advanced Research (CIFAR), Toronto, ON M5G 1M1, Canada; 8 NVIDIA, 431 King St. W 6th, Toronto, ON M5V 1K4, Canada

**Keywords:** large language model, quantum computing, self-driving laboratories, superconducting quantum processors, qubit calibration

## Abstract

Fully automated self-driving laboratories promise high-throughput, large-scale scientific discovery by reducing repetitive labor. However, they require deep integration of laboratory knowledge, which is often unstructured, multimodal, and hard to incorporate into current AI systems. This paper introduces the “k-agents” framework, designed to support experimentalists in organizing laboratory knowledge and automating experiments with agents. The framework uses large-language-model-based agents to encapsulate laboratory knowledge, including available operations and methods for analyzing results. To automate experiments, execution agents break multistep procedures into agent-based state machines, interact with other agents to execute steps, and analyze results. These results drive state transitions, enabling closed-loop feedback control. We demonstrate the system on a superconducting quantum processor, where agents autonomously planned and executed experiments for hours, successfully producing and characterizing entangled quantum states at human-level performance. Our knowledge-based agent system opens new possibilities for managing laboratory knowledge and accelerating scientific discovery.

## Highlights

• Language-model agents learn and apply laboratory knowledge
• Agents autonomously conduct complex quantum experiments
• System enables closed-loop feedback for experiment control
• Performance matches expert scientists in quantum calibration

## The bigger picture

Laboratories that can operate autonomously have the potential to accelerate scientific discovery, but they face a challenge: how to embed the knowledge that human researchers rely on when designing, running, and interpreting experiments. Our work addresses this challenge by introducing a language-model-driven agent framework that captures and operationalizes laboratory knowledge. This enables autonomous coordination, decision-making, and analysis. When our system was demonstrated in the complex domain of quantum computing, it autonomously performed multistep experiments and achieved outcomes comparable to those of expert researchers. This approach provides a scalable path toward more intelligent, adaptable laboratory automation, opening new opportunities for faster, more accessible scientific breakthroughs across experimental sciences.

## Teaser

This study introduces a language-model-driven agent system that captures and applies laboratory knowledge to autonomously plan, execute, and analyze experiments. When the agent system was demonstrated on quantum computing tasks, the agents achieved performance on par with expert researchers. By enabling intelligent coordination and decision-making in complex lab settings, this approach paves the way for more flexible, scalable automation in experimental science.

## Introduction

Automating laboratory experiments has the potential to accelerate scientific discovery by closing the loop between experimental execution, artificial intelligence (AI), and human-in-the-loop decision-making.^1^ Although it is increasingly common in laboratories that experiments can be implemented through programming interfaces,^2^^,^^3^ automating experiments still requires detailed laboratory knowledge to set the parameters of each experiment, interpret the results of the experiment, and execute complicated experiment workflows. Traditional methods of automating experiments require human experts to translate their knowledge into code for machine execution.^4^^,^^5^^,^^6^^,^^7^^,^^8^ However, the knowledge required for laboratory work has increased significantly over the past decades due to the growing complexity of the experimental apparatus, which now involves more intricate technical details for operation. This makes translating the required knowledge and maintaining consistency an increasingly significant human effort, which compromises the efficiency brought about by automation. In addition, the multimodal and complex nature of laboratory knowledge also makes it challenging to develop automated programs that can perform these tasks at a level comparable to that of humans.

The advent of large language models (LLMs) and multimodal LLMs has sparked new hope for more efficient experiment automation due to their universal ability to process text and image information.^9^^,^^10^^,^^11^^,^^12^^,^^13^^,^^14^^,^^15^ Although limited by the length of their context windows, LLMs offer the hope of efficiently understanding laboratory documents, performing analyses, generating code, and interpreting experimental images. The development of multiagent systems has further enhanced LLMs’ capabilities by integrating multiple LLM-based agents to work together on a complex objective, improving both accuracy and scalability.^16^^,^^17^^,^^18^^,^^19^^,^^20^^,^^21^^,^^22^ The merits of LLM-based agents make them well suited to assist laboratory automation without the need for extensive human intervention. However, several challenges remain. First, much of the media, such as code and documents, that carry laboratory knowledge are proprietary and inaccessible online, meaning that they are unlikely to be included in the training data of publicly available LLMs. In addition, the amount of text that includes such specialized laboratory knowledge is often insufficient to effectively fine-tune an LLM.^23^ Furthermore, laboratory knowledge is usually dynamic and frequently updated, making fine-tuning models impractical, as it is often hard to rewrite the knowledge in LLMs.^24^^,^^25^^,^^26^ As a result, incorporating laboratory-specific knowledge into LLMs remains difficult. Recent work on retrieval-augmented generation (RAG) offers a foundation to equip LLM-based agents with larger-scale knowledge without fine-tuning.^27^^,^^28^^,^^29^ However, laboratory knowledge is usually heterogeneous and multimodal, making it challenging to apply standard RAG methods directly.^4^^,^^8^^,^^30^

Despite challenges, pioneering efforts to develop automated LLM-based agents to carry out experiments are already underway.^31^^,^^32^^,^^33^^,^^34^ For example, in Boiko et al.^31^ and Bran et al.,^32^ researchers equipped LLM-based agents with tools that allowed them to acquire professional knowledge from the expert-designed tool kit and even the internet. These agents were then capable of performing chemistry experiments on the basis of this knowledge. However, in general, previous work has lacked a scalable memory system and relied on keeping the conversation history when calling LLMs. This inherently restricts the agents’ ability to automate long-duration tasks involving many sequential steps, as most current LLMs exhibit diminishing performance as the input length increases.^35^^,^^36^^,^^37^ This construction also prohibits further extensions of the system, such as supporting the handling of scientific plots.

**Figure 1:**
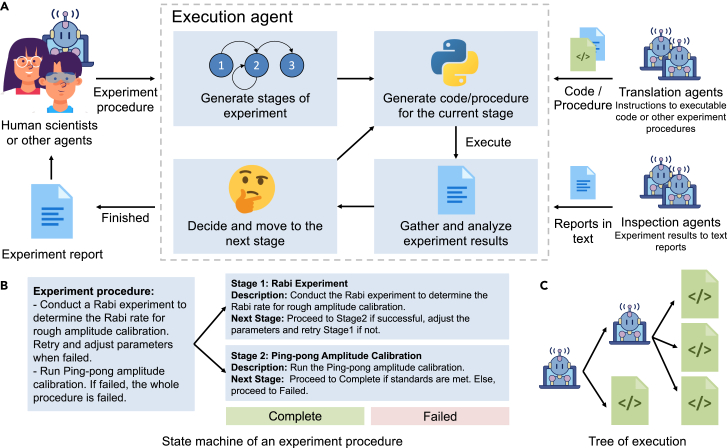
Overview of the “k-agents” framework and agent-based state machine architecture (A) Overview of the k-agents framework. Given a procedure in natural language, the execution agent first decomposes it into an agent-based state machine, which contains experiment stages. Each stage holds an independent experiment instruction to be translated with the translation agents. The transition between stages is driven by the generated transition rules and the reports from the inspection agents. (B) Agent-based state machine of instructions. The figure demonstrates how an experiment procedure can be decomposed into an agent-based state machine. The decomposition divides experiment procedures into experiment stages that hold single instructions along with a transition rule for deciding the next stage. (C) Tree of translation. The translating of instructions can be viewed as an expansion of a tree, in which the translation results are represented as the child nodes. An instruction can be translated as a simple experiment (code node) or an experiment procedure (agent node) that needs further execution and translation with another execution agent.

In this work, we introduce “k-agents” (see Figure 1), a knowledge-based multiagent system designed to automate experiments, particularly those requiring large-scale, multimodal laboratory knowledge and complex workflow. We developed tools for users to transfer their knowledge, creating LLM-based knowledge agents to manage laboratory knowledge without fine-tuning. These knowledge agents can help operate laboratories by holding knowledge ranging from single experiments to complex procedures and how to inspect the results of each experiment. These agents are designed to be activated selectively, allowing the system to scale efficiently as the number of agents increases. In order to fully exploit the knowledge agents, we further introduce the “execution agent,” which is responsible for coordinating the knowledge agents, gathering and filtering knowledge, generating code (scripts) to operate the laboratory, and controlling the progress of complex experiment procedures with intelligent closed-loop feedback planning. To handle multistep experiments, the execution agent decomposes complex procedures into independent experiment stages and creates an agent-based state machine. The agent-based state machine differs from a traditional state machine in that state transitions are determined by agents, rather than by rigid deterministic rules. This state-machine-based approach minimizes the experimental history that needs to be loaded into LLMs, making it feasible to conduct long-duration experiments at a human-like level of performance. The agents are created by prompting a language model on a distinct context, and the details of the prompt construction can be found in Note S2.

As a demonstration, we applied our framework to automate the calibration and characterization of single- and 2-qubit gates on our superconducting quantum processor. Superconducting qubits have become one of the most widely adopted platforms for quantum computing, with recent advancements pushing the scale to hundreds of qubits, along with active quantum error correction.^38^^,^^39^^,^^40^^,^^41^ As these systems grow in complexity, calibrating the operations of hundreds of qubits has emerged as a substantial bottleneck. Given this rapid progress, it is timely and essential to study automation solutions that can support the scalability and performance requirements of large-scale superconducting quantum devices. In addition, our framework demonstrates its ability to conduct custom experiments. Specifically, preparing the Greenberger-Horne-Zeilinger (GHZ) state requires the calibration of multiple qubits to execute the desired quantum operations. We selected this experiment to demonstrate the capabilities of our automation technique in generating an entangled quantum state and evaluating its fidelity. We propose that this framework could be adapted to other fields as a model for future research and industrial methodologies.

### Knowledge agents

We define “knowledge agents” as AI agents whose performance is measured by their ability to receive and transfer knowledge. For instance, an agent qualifies as a strong knowledge agent if it accepts knowledge from natural language inputs and responds correctly to related queries using natural language. Developing more advanced knowledge agents offers significant advantages. First, a more general ability to accept knowledge reduces the effort needed for humans to translate knowledge into computer-friendly formats. Furthermore, the ability to transfer knowledge facilitates more rigorous testing of agents^42^ and avoids treating the system as a black box, improving the general trustworthiness of AI systems.

Prior works^31^^,^^32^^,^^33^^,^^34^ have limitations in scalability, which constrain their ability to manage complex tool sets and finish tasks that require many steps. These systems typically construct a single prompt that embeds both task instructions and the entire tool set. However, as shown in Yao et al.,^43^ Wang et al.,^44^ and Barres et al.,^45^ the performance of these systems can degrade significantly as the length of input to the LLM, including the list of tools and history of action, increases. For example, in Barres et al.,^45^ the authors demonstrate that the performance of LLM agents can decrease to nearly zero when more than seven actions are required for the task in their setup.

To address some of these issues, works such as Boiko et al.^31^ introduce modular architectures that allow the developer to manually divide tools into components. Additionally, they rely on RAG^31^^,^^32^ for data access, which often screens and retrieves documents based on relevance rather than a deeper understanding of their structure.

To resolve the above issue, we introduce the k-agents framework, which provides tools to implement the aforementioned bidirectional knowledge transferability and the scalability toward a large number of tools and long-horizon tasks. In k-agents, we implement user-friendly interfaces that enable users to encapsulate knowledge within different LLM-based agents. These agents can then transfer their knowledge to the users and other agents in the format of natural language with LLMs. Our framework has a scalable memory architecture with an agent-based retrieval mechanism. This architecture enables good performance while enabling the required context length and token count to grow more slowly as the memory size grows. This enables agents to dynamically interact with a large, indexed memory of procedural knowledge and existing experiments. During retrieval, the agents conduct the reflection and selection processes to improve the accuracy and contextual relevance of retrieved information. This design allows our framework to support more complex and long-duration tasks.

#### Code translation agent

**Figure 2:**
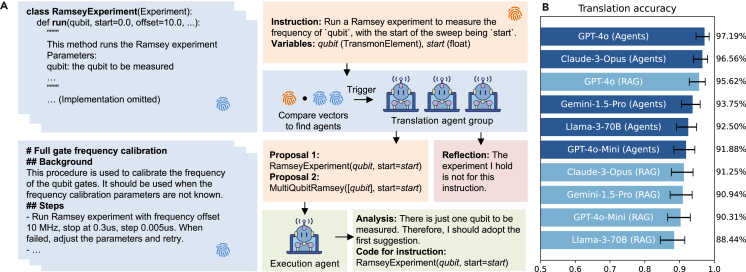
Translation agents architecture and performance benchmarking (A) Translation agents. Translation agents are responsible for translating an incoming instruction into executable code. The agents will be activated when their characterizing vectors (blue fingerprints) have a significant overlap with the instruction’s vector (orange fingerprint). The activated agents will try to translate the instruction based on their knowledge. If the translation agent deems the translation as valid, the result will be output to the execution agent for a final selection and execution. (B) Accuracy of instruction translation. We prepare a test set containing 80 instructions from eight experiments to be translated. We compare k-agents (marked as Agents), which uses Algorithm 1, with standard RAG methods (marked as RAG), which directly load the signature of the experiment class into the prompt. In our setup with 17 code translation agents, we found that k-agents demonstrates better performance than standard RAG methods in choosing the correct experiment class. Further, k-agents can support heterogeneous translation agents working together, which is hard to achieve with standard RAG methods. The error bars are estimated using the Wilson score confidence interval for binomial proportions. Details of this benchmark can be found in Note S3.

The first kind of knowledge agent in k-agents is the “code translation agent,” which is designed to accept and apply the knowledge of the available experiment interfaces that can be called by code. Here, the term “experiment” stands for not only a direct experiment on an instrument but any general action that is available in the laboratory. It can also be a call to a tool or even another agent. Given instructions in natural language, these agents are responsible for translating natural language instructions into the corresponding code. To help the users of k-agents construct code translation agents, we provide an abstract Python class that allows users to define the set of experiments available in the laboratory explicitly. The users can then define all possible experiment interfaces, ensuring that the AI system can access and utilize them. As illustrated in Figure 2A, human experimentalists are expected to document background knowledge, required parameters, and implementation code for each experiment in the run method of the subclass representing the experiment. After indexing all available experiment classes, k-agents will construct a code translation agent for each of the classes.

#### Procedure translation agent

Experiment procedures in laboratories can involve complex workflows. These workflows may require experimentalists to determine a sequence of experiments based on the results of the experiments executed. Traditionally, this knowledge has been maintained either through unstructured documentation or by relying on the memory of the experimentalists themselves. To address this, as shown in Figure 2A, k-agents introduces a standardized format to store examples of how to implement instructions using multistep experiment procedures. These examples are then used to produce another kind of knowledge agent that we call the “procedure translation agent,” which is capable of translating an instruction into its corresponding procedure based on stored examples. Similar to code translation agents, procedure translation agents also output code. The code will call an execution agent to execute the procedure, which we will introduce later.

#### Inspection agent

**Figure 3:**
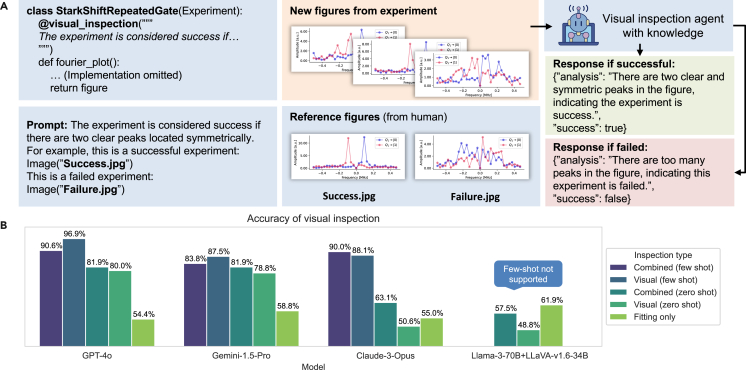
Visual inspection agents and performance benchmarking (A) Visual inspection agent. Our framework introduces visual inspection agents by attaching decorators to the member methods of the experiment class that produce figures. The decorator accepts a prompt as input, and users can add example figures to the prompt. Whenever the experiment is carried out, the execution agent can call the inspection agents to generate a text-based report based on the outcome figure and their knowledge (prompts). (B) Performance benchmarking of visual inspection. We compare the performance of the visual inspection method in determining whether a certain experiment is successful or not. We added three types of inspection agents. Visual agents with a text description of success criteria (zero-shot), visual agents with text descriptions and example figures (few-shot), and fitting agents who provide reports based on fitting results. We test the accuracy of inspection on each type of agent. We also combine the results of fitting agents and visual agents in the “combined” setup. We found that providing example figures significantly improves accuracy. Additionally, we found that reports synthesized from multiple inspection agents can give better accuracy in some cases.

A major burden for experimentalists has been the need to wait for experimental results and decide on the next step based on them. In many scenarios, experimentalists must analyze figures to assess the success of an experiment and determine the next steps. In the k-agents framework, we introduce inspection agents that have the knowledge needed to evaluate the results of each experiment. These agents will be called after the execution of each experiment to analyze its outcomes. We provide an interface to inject knowledge for each function that produces experiment figures. This feature is implemented as a Python decorator. In the decorator, the users can add instructions for how to analyze the figure. Besides, as illustrated in Figure 3A, our interface allows users to add example figures to help others understand the new figures. Based on the knowledge from the interfaces, we construct inspection agents equipped with multimodal LLMs to inspect and analyze new experiment figures and output text-based reports based on their knowledge.

### Execution agent

We introduce the execution agent to coordinate knowledge agents and manage complex experiment procedures. This agent takes experiment procedures written in natural language as input and driven by a text-only LLM. Since the selection of the next experiment in a procedure could depend on the results of previous experiments, we design the execution agent to first decompose the procedure into a “state machine,” in which each state represents a distinct experiment stage of the procedure (see Figure 1C). Each stage of the experiment is assigned a single instruction that contains only one experiment or subprocedure. As an agent-based state machine, there is also a transition rule at each stage in natural language that determines how to select the next stage based on the result of the experiment.

By constructing the agent-based state machine, the execution agent transforms the task of executing the procedure into executing the agent-based state machine. The execution is illustrated in Figure 1A. Starting from the initial stage, at each stage, the execution first translates the instruction at the stage into executable code with the assistance of translation agents. To increase the accuracy and efficiency of the translation, the execution agent will activate only translation agents related to the context. The relevance score will be calculated based on the embedding similarity between the context and the description of the translation agents. The execution will select a reasonable response from the translation agents and execute the translated code. After executing the code, the agent gathers a natural language report from inspection agents and, based on the report and the transition rules, determines the next stage to transition to. If the reports suggest updating the parameters in the new stage, the execution agent will also pass the update to it. This state-machine architecture enables the execution agent to focus on executing each stage of the procedure efficiently without needing to monitor the entire experimental history when deciding on the next experiment.

## Results

In this section, we demonstrate how the k-agents framework can be applied to the calibration and operation of a superconducting quantum processor. Superconducting circuits provide a popular physical platform for building quantum information processors. These circuits are fabricated on a chip using nanofabrication techniques. The circuit components operate under quantum mechanical principles at millikelvin temperatures, forming non-linear quantum resonators, which can be excited similar to atoms using microwave signals. Information can be stored in the quantum states of these resonators, which serve as qubits on the processor.^46^^,^^47^^,^^48^^,^^49^ To control these qubits, external electronics connected to the chip generate precise microwave signals. These signals must be carefully calibrated to accurately set the operational parameters, which are crucial for executing logical quantum gates. Typically, the parameters include the shape of the pulse (the duration and amplitude at a certain time *t*) and the initial phase and the frequency of the pulse. For a 2-qubit gate, multiple pulses may be required to send at the same time to generate the entanglement.^48^^,^^50^^,^^51^

A significant challenge in superconducting circuit research is identifying these optimal operation parameters. Although automated scripts can determine some parameters, they still require customization by scientists based on different hardware setups. Moreover, programmatically validating the correctness of the results of these scripts is usually difficult to implement. Scientists must continuously monitor experimental status and adjust the calibration scripts to optimize operational parameters. Moreover, these parameters drift over time and occasionally require recalibration. This process is time consuming and repetitive, often not yielding direct results for physical research, yet it is essential for conducting superconducting circuit research. This calibration process is complex and labor intensive, leading to significant challenges for scaling quantum computing systems. These difficulties are not limited to the superconducting circuit platform, but also extend to ion traps,^52^^,^^53^^,^^54^ spin qubits,^55^^,^^56^^,^^57^^,^^58^ and Rydberg atom systems.^59^^,^^60^^,^^61^ Moreover, the techniques developed for these tasks have broader implications beyond quantum computing, which has great potential to accelerate scientific and technological research in general.

The above challenges fit the targeting scenario of the k-agents framework. We adapt our existing control software stack to the k-agents framework, which enables monitoring experimental results, recommending further parameter adjustment, and executing automated calibrations and experiments. To demonstrate its capabilities and assess effectiveness, we conducted three experiments. Before moving on to the hardware demonstration, we first benchmarked the performance of k-agents in the context of superconducting qubits. We adopted the LeeQ software,^62^ originally used to control superconducting quantum processors in the lab, to work with the k-agents system and provided it with additional multimodal information to determine the success or failure of the experiments (see Note S4 for more detail). First, we benchmarked the translation process using different LLM models, comparing the typical RAG method with our approach. Our results show that the GPT-4o model has the best performance and archives an accuracy of 97%, which we consider sufficient for practical application (see Figure 2B). In practice, when code generation fails, producing unusable code or syntax errors, we prompt the agent to retry, further improving the success rate. We also evaluated the inspection agents to determine if they could accurately identify the success or failure of experiments. We found that the best performance was achieved when using inspection agents constructed with example figures (see Figure 3B).

Following the successful benchmarking results, we designed the experiments to further evaluate the performance of k-agents on real hardware. First, we demonstrate fully automated recalibration of single-qubit parameters. Second, we showcase the automated discovery and calibration of 2-qubit gates on our platform. Third, we highlight the AI agent’s ability to generate quantum states based on natural language descriptions, including the creation of a GHZ state across 3 qubits, utilizing previous calibrations.

Our experimental platform is a 16-qubit superconducting quantum processor configured in a four-by-four square lattice connectivity layout. The characterization details of this device have been reported in Note S6. The qubits employed are standard fixed-frequency transmon qubits with coaxial geometry.^63^^,^^64^ For the demonstration of our framework, we selected a subset of three adjacent qubits. In superconducting qubit systems, 2-qubit gates usually have to be implemented between adjacent qubits, and the crosstalk and noise are more significant. Our demonstration using an adjacent 3-qubit subset is consistent with current hardware constraints and design practices. Although it involves only a few qubits, this setup reflects the typical conditions of academic labs, where small-scale experiments suffice for exploring quantum phenomena and improving qubit performance. For further details about the hardware setup, please refer to Note S6.

**Figure 4:**
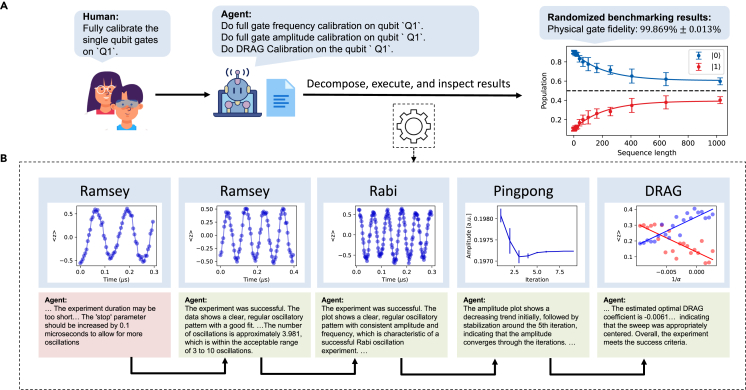
Automated single-qubit parameter recalibration driven by k-agents (A) The human scientist instructs the k-agents framework for calibrating the single-qubit gate parameters. Based on the reference documents provided in natural language, k-agents successfully breaks the calibration into four steps, followed by the randomized benchmarking experiment to characterize the single-qubit gate fidelity. (B) In this example, k-agents finds that the first Ramsey experiment had not collected enough oscillations to estimate the frequency. Therefore, it repeats this experiment by increasing the experiment time. The following experiments all pass the success criteria of the k-agents. In the end, k-agents implements the randomized benchmarking and reports the gate fidelity.

In the first experiment, we demonstrate automated calibration and benchmarking of a single qubit with k-agents. To facilitate the calibration process, we provided the agent with a document detailing the general procedure for single-qubit calibration in natural language. At the start of the experiment, we manually adjusted the initial parameters to deviate from their optimal values. Based on the document, the execution agent first decomposed the procedure into two distinct stages: calibration and benchmarking. The calibration stage was further broken down into multiple steps, in which the agent sequentially adjusted the qubit’s frequency, amplitude, and DRAG (derivative removal by adiabatic gate) parameters. If a failure occurred during the procedure, the step would be retried. After several unsuccessful attempts, it would revert to the previous stage to try again. This process is driven by the agent to emulate the typical behavior of a human scientist who implements the calibration process. We observed that the agent was able to perform transition correctly between stages based on the experiment result and found the acceptable parameters. At the benchmarking stage, the agent successfully performed a randomized benchmarking on the single qubit and validated its fidelity (see Figure 4).

Following the single-qubit calibration experiment, we demonstrated the automated discovery of 2-qubit gate parameters. Specifically, we focused on the siZZle (Stark-induced ZZ by level excursions; ZZ is an interaction in which two qubits influence each other’s states) gate,^65^^,^^66^^,^^67^^,^^68^ which can be used to create entanglement between two fixed-frequency superconducting qubits. By driving both qubits off resonance at the same frequency simultaneously, the ZZ term in the qubits’ Hamiltonian is altered compared to when the qubits are undriven. Leveraging this effect, we constructed a pulse sequence with calibrated amplitude and frequency to adjust the ZZ interaction strength, followed by fine-tuning the pulse duration to achieve precise qubit entanglement. The most challenging part of the process was the search for optimal driving amplitude and frequency, while the pulse duration calibration was relatively straightforward, accomplished programmatically by measuring the ZZ interaction rate during the drive. In this experiment, the AI agent autonomously identified a set of working parameters for the siZZle gate.

For a human scientist, searching these parameters typically requires starting with empirical knowledge and measuring the ZZ interaction strength at specific driving frequencies and amplitude. The search process then proceeds by adjusting the frequency and amplitude based on the experimental outcomes. When an experiment succeeds, we often increase the pulse amplitude to enhance the ZZ interaction rate or adjust the driving frequency closer to the qubit’s transition frequency. Conversely, when an experiment fails, we either decrease the amplitude or move the frequency away from the transition frequency. There are three regions that need to be explored: below both qubits’ transition frequencies, between the two, and above both qubits’ transition frequencies. We inject this empirical knowledge of parameter selection into two LLM-based agents that are accessible to the experiment history for what parameters have been tried. By wrapping these agents into two special experiments, the execution agent can call them to get the next frequency and amplitude to try. The parameter searching can then be formulated as an alternate calling to these special experiments and the experiments for testing the proposed parameters.

For each parameter pair (frequency and amplitude), we measured the ZZ interaction rate by performing Hamiltonian tomography on the ZZ term using two methods: continuous-time tomography and repeated-gate tomography. The latter method, which accounts for the effects of the pulse’s rising and falling edges, yielded more accurate ZZ interaction measurements per pulse.

The most challenging aspect was determining the success of each experiment. To address this, we plotted the Fourier transform of the Hamiltonian tomography results and let an inspection agent decide whether the experiment was successful, where the agent is equipped with a few-shot visual knowledge about the success criteria. Furthermore, we monitored the state of the control qubit, plotting its expectation value of ⟨Z⟩, and tasked another inspection agent with ensuring that it remained stable during the driving process, avoiding any unwanted excitation.

**Figure 5:**
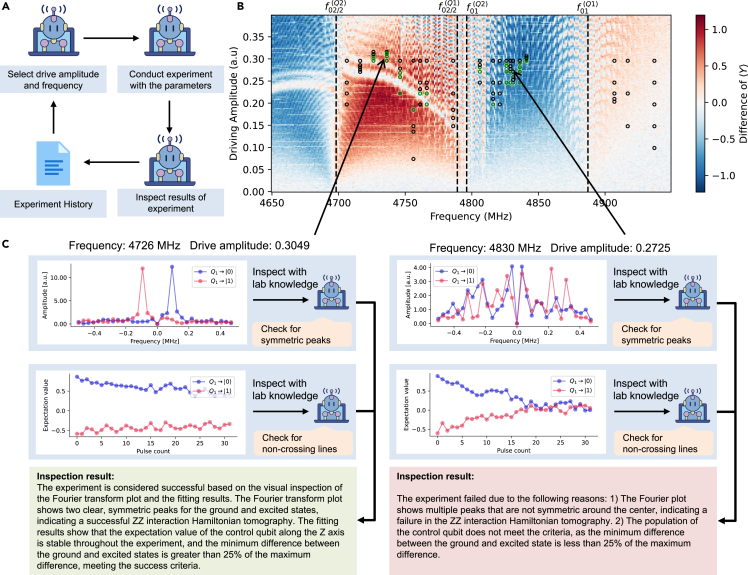
Two-qubit gate parameter discovery driven by k-agents (A) The workflow of the discovery process, where k-agents continuously reads the history of previous experiments and suggests the next parameter set likely to succeed. (B) The set of discovered parameters and the corresponding parameter search space. The background indicates the ZZ interaction strengths visualized from data collected during an overnight experiment of which the agent has no prior knowledge. The green circles denote locations where the agent identified an acceptable parameter set, while the black regions indicate failed attempts. For more details on the search for siZZle gate parameters, refer to Note S5. (C) Examples of figures observed by the agent, along with its response, illustrating a typical success case and a failure case, respectively.

We ran the experiment for 3 h, limiting the execution agent to carrying out 100 experiments to search for parameters and test a maximum of 20 frequencies. By the end of the experiment (see Figure 5), the agent successfully identified an optimal set of parameters (frequency 4,726 MHz and amplitude 0.3049).

**Figure 6:**
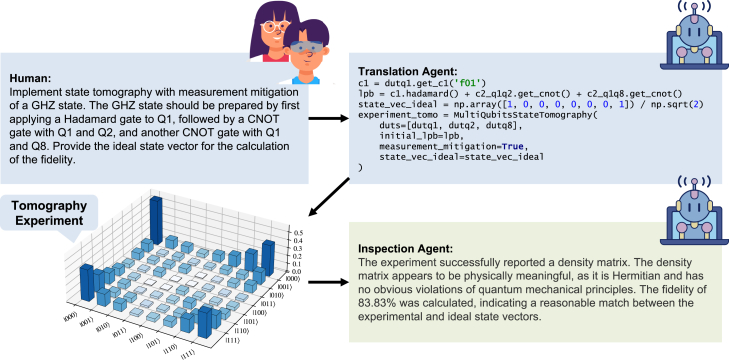
Automation of the GHZ state generation and tomography with k-agents First, the instructions are provided by human scientists. Then, the code is generated by the translation agent to implement the experiment. The density matrix plot of the generated GHZ state is constructed from the experiment result. Finally, a report in natural language is generated by an inspection agent.

Finally, we demonstrate the ability of the AI agent to carry out experiments based on natural language instructions. After successfully calibrating the gates, we instructed the execution agent to perform state tomography for a GHZ state. Additionally, we requested the agent to perform process tomography for the 2-qubit gate parameters it had calibrated. The agent successfully implemented this experiment and reported the state fidelity (see Figure 6).

## Discussion

In this study, we introduce the k-agents framework for creating laboratory-related knowledge agents and demonstrate its capability to automate experiments. We applied the system in a real-world setting, successfully demonstrating the ability of k-agents by performing single-qubit and 2-qubit gate calibrations as well as generating and characterizing a GHZ state on a quantum processor based on human instructions. The k-agents succeeded in orchestrating the experiments, interpreting the multimodal experimental results, and guiding the execution of the experiment with closed-loop feedback. These achievements suggest that our system is a valuable tool for research groups working with superconducting quantum processors, with the potential for broader applications in research automation across other fields. Additionally, we also applied two benchmark experiments on k-agents. We found that our agent-based translation of natural language instructions outperforms standard RAG approaches in terms of accuracy. By our benchmark of the inspection agents, we showed that current multimodal LLMs have the ability to analyze experiment figures based on human instructions, especially when provided with example figures.

While methods such as rule-based systems, reinforcement learning, and Bayesian optimization have been used for calibrating quantum devices, our approach is fundamentally different. First, the k-agents framework is designed to automate entire experimental procedures, rather than focusing on the tuning of specific parameters. Optimization methods like reinforcement learning and Bayesian optimization typically require extensive manual tuning and carefully crafted reward functions and are limited to narrow, predefined tasks. In contrast, our framework provides a more general, interpretable, and adaptable solution, particularly well suited to dynamic and evolving quantum laboratory environments. Moreover, unlike traditional rule-based systems that depend on rigid, hand-coded logic, the k-agents framework uses multimodal prompts (natural language and images) and LLMs to enable more flexible and efficient automation.

One of the strengths of the k-agents framework is its approach to knowledge representation. It requires no more effort than maintaining standard laboratory documentation and fits naturally into routine protocol management. While we acknowledge that adopting any new framework involves some onboarding and training, the setup effort is comparable to preparing laboratory manuals or protocols for new PhD students or junior staff. Once standard documents already exist—which is common practice in most laboratories—our agents can be configured with minimal additional work.

While our framework shares conceptual similarities with prior agent systems like ChemCrow^32^ and Voyager,^69^ it introduces several important distinctions that enhance agent-based AI for laboratory automation. First, our framework is specifically tailored for quantum computing laboratories, where experiment automation involves breaking down long-horizon workflows and dynamically adjusting parameterized experiments by analyzing previous results. In contrast, ChemCrow targets open-ended challenges in general chemistry, focusing on relatively straightforward, short-horizon tasks that require less iterative reasoning and fewer reexecutions. Second, unlike Voyager, which utilizes large internet-scale corpora about Minecraft that has already trained into LLMs, our system operates effectively without depending on such extensive external data, making it better suited to laboratory environments. Additionally, Voyager develops agent skills through simulated environments—a resource not always available in laboratory settings—whereas our k-agents framework emphasizes practical application by building on existing laboratory code bases. These distinctions reflect our commitment to improving the practicality and scalability of agent-based AI for real-world scientific experimentation.

However, the k-agents framework has some potential weaknesses that make full automation challenging. First, our method relies on clean and well-formatted code bases and procedure documents. However, such structured knowledge may not always exist, and transforming existing knowledge could take considerable human effort. Future research on structuralizing unstructured documents and code bases is still needed to completely automate knowledge translation. A possible direction of research could involve enabling the framework to generate structured code and procedures based on unstructured project files and human inputs. Second, using LLM, the execution of k-agents exhibits higher latency than that of traditional automation methods that do not require LLM-based planning and translation of knowledge at runtime. We note that this problem can be well mitigated by caching the response of LLMs and using the same response whenever the input is the same.

In addition, our current implementation of execution agents within the agent-based state machine does not support fine-grained, interruptible execution; it allows only for termination of the entire program in response to external signals or emergency conditions. However, we acknowledge that in quantum systems, it would be beneficial to provide an interactive mechanism for human scientists to monitor and intervene in the experiment’s progress. Addressing this limitation is a priority for future development, and we plan to explore the integration of real-time interrupt mechanisms, hardware-level hooks, and human-in-the-loop safety protocols.

We monitor the cost of the k-agents system. During the parameter search for the 2-qubit gate, we observed that the LLM used 1,373,207 input tokens (including image tokens) and 168,039 output tokens in 3 h, costing less than US$5.00. We believe that there is potential for further optimization to reduce these costs. Regarding time efficiency, despite network delays, LLMs currently take longer than humans to perform inspections. The inspection agent utilizes multimodal LLMs and requires a few seconds to evaluate each experimental result, with an inference time comparable to that of the standard LLM agents without the multimodal ability. However, LLMs tend to be more efficient than humans in generating code. Therefore, overall efficiency is comparable.

The use of superconducting quantum processors simplifies some challenges, such as safety control and error tolerance. In our system, the risk of the agent executing code that causes significant damage or unacceptable costs is very low. If it fails, we can simply allow it to try again. However, this may not be the case in other applications. Prior research^1^^,^^70^^,^^71^ has focused heavily on safety control, and this is an area we plan to explore further in the future.

## Methods

### Details on the knowledge agents developed

#### Definition of knowledge agents

The concept of knowledge agents is defined based on the work proposed by Zhang et al.,^15^ in which an agent’s learning ability is defined as its ability to internalize knowledge given related inputs. A knowledge agent can then be interpreted as an agent with the ability to learn (i.e., acquire knowledge) and to apply the learned knowledge to respond to other agents (i.e., transfer knowledge). A bare LLM can be regarded as a knowledge agent as it is able to absorb knowledge from its training data. However, it is difficult for LLMs to internalize laboratory documents and codes as their knowledge because of the current pitfalls in model editing.^24^ Therefore, we regard bare LLMs as weak knowledge agents compared to the knowledge agents we proposed, which are good at accepting knowledge from laboratory documentation.

#### Translation agents

In k-agents, we included two types of translation agents: “code translation agent,” which translates instructions into code, and “procedure translation agent,” which translates instructions into experiment procedures that may contain multiple institutions. Each translation agent is designed to process only instructions that are very close to the description of an existing experiment. However, by recursively translating the instructions, the whole system can achieve higher generalizability.

##### Initialization

Each code translation agent is directly constructed from a class of experiments. Similarly, procedure translation agents are also constructed from the structured documents of procedures mentioned above. The document translation agent will try to translate the instructions that resemble the title of the experiment item into code that invokes an execution agent to execute the corresponding procedure.

##### Activation

Many translation agents might exist based on the complexity of the experimental system. However, not all translation agents are needed to translate a certain instruction. Therefore, we designed the system to use embedding similarity and to activate only the agents related to the instruction to be translated. When generating the agent, we use LLMs to generate a series of natural language instructions {I} that the agent should translate. We further calculate the embeddings $\left\{\vec{E}\right\}$ of these instructions. When there is an incoming instruction with embedding $\vec{E_{I}}$, the score *S* of a translation agent is calculated by the maximal inner product between $E_{I}$ and the vectors in $\left\{\vec{E}\right\}$:

(Equation 1) $$S=\max_{\vec{E_{i}}\in \left\{\vec{E}\right\}}\vec{E_{I}}\cdot {\vec{E_{i}}}^{†}\text{.}$$

##### Code translation

When activated, the code translation agent attempts to translate the instruction into executable code. The Python class signature for the experiment and the instruction will be passed to an LLM to generate a code translation based on the class. We employ two strategies to improve the translation accuracy throughout this process. First, we implement the chain-of-thought (CoT)^72^ strategy to improve performance. Before generating code, the LLM is first asked to provide a paragraph of analysis on how to approach the translation. This helps guide the LLM in retrieving key points from the context and making useful inferences. Next, we adopt a self-reflection strategy to improve the accuracy. The agent will determine whether the instruction should be translated using the experiment class based on the previous analysis. This step selects out cases where the class is not suitable for the instruction, helping to avoid hallucinations. If the agent concludes that the instruction should be translated into code using the class, it proceeds to generate a candidate code snippet. This candidate code is then sent to the execution agent for comparison with other potential candidates.

##### Procedure translation

Our procedure translation agents are designed similarly to code translation agents. If an agent deems an incoming instruction matches the procedure it holds, it will try to rewrite the procedure title to suit the instruction. A code snippet that calls an execution agent to execute the rewritten title will then be generated and sent to the execution agent as a code candidate.

##### Generalizability

In both types of translations, the agents generate new code and new instructions based on the input instruction and therefore generalize the knowledge they have. However, this generalizability is well controlled, as translations that deviate too much from the agent’s knowledge will be decided as improper by the strategy we described. This limited generalizability mitigates the negative effect of the LLM’s knowledge from the public corpus that is not suitable for a private experimental setup.

### Details on execution agents

In k-agents, the execution of experiments is controlled by the execution agents. The execution process involves decomposing the incoming procedure into experiment stages, translating the instruction at each stage into code by translation agents, executing the generated code, and analyzing the experiment results to determine the next step.

#### Instruction decomposition

When handling experiment procedures, we ask the execution agent to decompose the instructions into experimental stages and make an agent-based state machine. Each stage in the agent-based state machine contains a single step of the instructions that includes only one experiment or subprocedure to be translated by the translation agents. In addition, an experiment procedure might have a complex control flow. For example, selecting the next instruction to execute might depend on the result of the previous instructions. Therefore, at each stage, we also attach a prompt on how to select the next stage given the result of this stage.

The stage generation is done roughly in two calls to an LLM. First, we employ the LLM to extract a list of independent experiments from the procedure. This step removes all the sentences controlling the procedure’s progression and generates the instructions at each stage. Then, we add indices to the stages, such as stage 1 and stage 2, so their indices can be used to refer to them when generating the transition rules. We also add two special stages, COMPLETE and FAILED, to the list of stages. Finally, we provide the LLM with the list of stages and the original procedure, asking it to attach the transition rule for each experiment.

#### Translation candidate selection

When executing a stage, the execution agent activates the translation agents and gathers candidate code from them. Then, the execution agent synthesizes the code to execute on the basis of the candidates it receives. Specifically, all the candidate code will be input into an LLM with a prompt that asks the LLM to generate an analysis and the final code for the translation. This synthesis process has two merits. First, there might be two experiments with similar descriptions. The translation agents for them are likely to generate candidate code together. By providing them as candidates, we make it possible for LLM to analyze which solution is more suitable. Further, as the activating mechanism might not be perfect, there might not be any answer from the translation agents because the desired translation agent is not activated. In this case, we design the execution agent to try to activate more translation agents until a proper candidate appears. We summarize the translation process in Algorithm 1. When applying the algorithm in Section Results, we set $N_{k}=3$ and $N_{\max}=9$.

Algorithm 1 **Translation with self-reflection**

(1) Set the group of translation agents {A}, the number $N_{k}$ for the number of activated agents, and the number $N_{\max}$ for the maximal number of activated agents.
(2) Input the instruction *I* to be translated.
(3) Calculate the score of each agent in {A} by Equation 1.
(4) For each agent *A* whose score is ranked top $N_{k}$ in {A}, do the following:
  (a) Using LLM, check whether the instruction can be translated by the knowledge held by *A.* If so, generate the code *C* that implements the instruction. Else, output nothing.
  (b) Add *C* to the set of code candidates.
(5) If the set of code candidates {C} is empty, increase the number of $N_{k}$ by 2 and do the following: if $N_{k}<N_{\max}$, go back to step 2. Else, the algorithm fails.
(6) If {C} is non-empty, add the instruction and the set of code candidates {C} to the context of LLM and generate the code $C^{*}$ for *I.*
(7) Output $C^{*}$ as the result of translation.

#### Execution

Starting from the first stage, the execution agent executes the instruction in the current stage by executing the translated code with the help of the translation agents, as described in the previous section. After execution, the inspection agent generates a report of the experiment in natural language. Based on the report, the execution agent chooses the next state to transition to. Finally, after reaching the COMPLETE or the FAILED state, the execution agent will generate a report of the entire execution. We summarize the execution process in Algorithm 2.

Algorithm 2 **Execution agent workflow**

(1) Input the experiment procedure *P.*
(2) Using LLM, decompose *P* into experiment stages (states) {S} and transition rules {T} at each stage. The decomposition also specifies an initial state $S_{0}$ as the current state $S^{*}$. Two final states, COMPLETE and FAILED, are also in the S{}.
(3) At the current experiment stage $S^{*}$, do the following:
  (a) Translate the instruction contained in $S^{*}$ into code with Algorithm 1.
  (b) Execute the translated code.
  (c) Activate available inspection agents in the executed experiment and summarize the results from them with LLM, giving a summarized report *R.* Add $S^{*}$ and *R* to the experiment history.
  (d) Using LLM, decide the next stage to transition to, based on the transition rule $T^{*}$ of $S^{*}$ and the report *R.*
  (e) If the next stage is COMPLETE or FAILED, add it to the experiment history and break the loop (i.e., go to step 4). Else, set $S^{*}$ to be the next stage and go to step 3(a).
(4) Using LLM, summarize the experiment history and produce a report *R* for *P.*

We note that the execution agent also maintains a variable table. At the end of each experiment, variables might be injected from the experiment for the use of other experiments. The injection of variables can be programmed by the users when they implement the class for the experiment.

### Quantum processor hardware

The quantum processor used in this experiment consists of 16 coaxmon qubits arranged in a square lattice configuration. Coaxmon qubits, a variant of transmon qubits with coaxial geometry and off-chip wiring, have demonstrated high coherence, low crosstalk, and stable higher excited states.^63^^,^^64^^,^^73^^,^^74^

To enable the execution agents to conduct experiments, we integrated the k-agents framework with the LeeQ framework,^62^ customized software used for controlling superconducting quantum processors in the lab. The LeeQ framework, which has been independently used by human scientists, manages the composition, compilation, and optimization of quantum gates into executable instructions. These processed microwave sequences are then sent to the QubiC system, an electronic system that generates and processes microwave signals and connects to the quantum processor.

In practice, human scientists write code that predefines experiments and reuse it in different scenarios by modifying the arguments of these experiments. Typically, scientists manually adjust the parameters of these experiments until they achieve their desired outcome. We adapted these predefined experiments to be compatible with and indexable by k-agents. In total, the LeeQ framework includes more than 40 built-in experiments, of which we selected 17 for indexing by the k-agents. For further details on the experiments, please refer to Note S4.

## Resource availability

### Lead contact

Requests for further information and resources should be directed to and will be fulfilled by the lead contact, Alán Aspuru-Guzik (alan@aspuru.com).

### Materials availability

This study did not generate new unique reagents.

### Data and code availability

The source codes for k-agents can be found in Zhang.^75^ The codes for the experiments and benchmarks can be found in Cao et al.^62^

## Acknowledgments

The authors thank Yuning Zhang for insightful discussions. This project is supported by Schmidt Sciences, LLC. S.C. acknowledges support from Schmidt Science. P.L. acknowledges support from the 10.13039/501100000266EPSRC (EP/T001062/1, EP/N015118/1, and EP/M013243/1). M.B. acknowledges support from an 10.13039/501100000266EPSRC QT Fellowship grant (EP/W027992/1). A.A.-G. thanks Anders G. Frøseth for his generous support. A.A.-G. also acknowledges the generous support of 10.13039/501100000159Natural Resources Canada and the Canada 150 Research Chairs program. This research is part of the University of Toronto’s Acceleration Consortium, which receives funding from the 10.13039/501100010785Canada First Research Excellence Fund (CFREF).

## Author contributions

S.C.: conceptualization, investigation, methodology, software, validation, writing – original draft, and writing – review & editing; Z.Z.: conceptualization, methodology, software, validation, writing – original draft, and writing – review & editing; M.A.: investigation and writing – review & editing; S.D.F.: investigation and writing – review & editing; M.P.: investigation; M.B.: resources; P.L.: resources; A.A.-G.: supervision and project administration.

## Declaration of interests

A.A.-G. is a founder of Kebotix, Inc., a company specializing in closed-loop molecular discovery, and IntrepidLabs, Inc., a company using self-driving laboratories for pharmaceuticals. P.L. is the founder and chief science officer of Oxford Quantum Circuits Limited, a company developing superconducting circuit quantum computers.

## Declaration of generative AI and AI-assisted technologies in the writing process

During the preparation of this work, the authors used ChatGPT in order to check for grammar errors and typos and improve the clarity of the writing. After using this service, the authors reviewed and edited the content as needed and take full responsibility for the content of the publication.

## Supplemental information

Document S1. Figures S1–S4, Table S1, and Notes S1–S6

Document S2. Article plus supplemental information
