# Supplementary material for: Automating quantum computing laboratory experiments with an agent-based AI framework
Source: Patterns (N Y). 2025 Sep 23;6(10):101372. doi: 10.1016/j.patter.2025.101372 (PMC12546452; doi:10.1016/j.patter.2025.101372)
Supplement: Document S1. Figures S1–S4, Table S1, and Notes S1–S6 [file mmc1.pdf]

Patterns, Volume 6

## Supplemental information

# Automating quantum computing laboratory experiments with an agent-based AI framework

Shuxiang Cao, Zijian Zhang, Mohammed Alghadeer, Simone D. Fasciati, Michele Piscitelli, Mustafa Bakr, Peter Leek, and Alán Aspuru-Guzik

# 1 Supplemental Note 1: Details on knowledge representation

## 1.1 Representing knowledge of available experiments

In *k-agents*, laboratory experiments (and actions) are formulated as sub-classes of an abstract class Experiment. The abstract class provides a few interfaces to put the documentation and code of the experiment. We use the following example to demonstrate how the interfaces are used.

**An example of experiment class**

```
class SimpleRamseyMultilevel(Experiment):

    _experiment_result_analysis_instructions = """
    The Ramsey experiment is a quantum mechanics experiment that involves the measurement of oscillations
         in the quantum state of a qubit. Typically, a successful Ramsey experiment will show a clear,
         regular oscillatory pattern with an amplitude greater than 0.2. If less than approximately 3
         oscillations are observed, the experiment requires an increase in the time of the experiment. If
         more than 10 oscillations are observed, the experiment requires a decrease in the time of the
         experiment. The frequency of the oscillations should be less than the expected offset value.
    """

    def run(self,
            qubit,
            collection_name: str = 'f01',
            mprim_index: int = 0,
            initial_lpb: Optional[Any] = None,
            start: float = 0.0,
            stop: float = 1.0,
            step: float = 0.005,
            set_offset: float = 10.0,
            update: bool = True) -> None:
        """
        Ramsey experiment for estimating the qubit frequency or T2 Ramsey.
        Parameters:
            qubit: The qubit on which the experiment is performed.
            collection_name: The name of the frequency collection (e.g., 'f01').
            mprim_index: The index of the measurement primitive.
            initial_lpb: Initial set of commands, if any.
            start: The start time for the sweep.
            stop: The stop time for the sweep.
            step: The step size for the frequency sweep.
            set_offset: The frequency offset.
            update: Whether to update parameters after the experiment.
        """
        ...

    @visual_inspection("""
        Here is a plot of data from a quantum mechanics experiment involving Ramsey oscillations. Can you
             analyze whether this plot shows a successful experiment or a failed one? Please consider the
             following aspects in your analysis:
        1. Clarity of Oscillation: Describe if the data points show a clear, regular oscillatory pattern.
        2. Fit Quality: Evaluate whether the fit line closely follows the data points throughout the plot.
        3. Data Spread: Assess if the data points are tightly clustered around the fit line or if they are
               widely dispersed.
        4. Amplitude and Frequency: Note any inconsistencies in the amplitude and frequency of the
             oscillations. For a perfect experiment, the amplitude should be around 1, and the frequency
             should be close to the expected value. If the amplitude is smaller than 0.2, the experiment
             is likely considered failed.
        5. Overall Pattern: Provide a general assessment of the plot based on the typical characteristics
             of successful Ramsey oscillation experiments.
        Here is an example of a successful experiment:
        Image("succcess.jpg")
        Here is an example of a failed experiment:
        Image("fail.jpg")
        """)
    def plot(self):
        ...

    @text_inspection
    def fitting(self):
        ...
        return {
            "success": True,
            "analysis": "The experiment is successful based on the fitting results."
        }
```

The run method of the class holds the lower-level code that carries out the experiment. The docstring of this method holds the description of the experiment and the required arguments.

This information will be used when the corresponding code translation agent tries to interpret instructions into code with this class.

The results of many experiments are more intuitive if they are represented in figures. However, interpreting these figures requires domain-specific knowledge, which is beyond the scope of most multimodal LLMs. For this, we provide a function decorator visual_inspection to represent the knowledge of how to analyze the figure. The decorator accepts a prompt as a parameter and uses it to generate a visual inspection agent that keeps the prompt as its knowledge. When the experiment is finished, and the experiment results are available in the experiment object, visual inspection agents will be called with the newly produced figures. The visual inspection agent will put the figure into a multimodal LLM along with the prompt to produce the report. We additionally note that our decorator accepts few-shot prompting. All the patterns Image("*") in the parameter, where * is a path to an image, will be automatically detected and replaced by the corresponding image located at the path.

Besides visual methods to analyze experiment results, we also provide an interface to produce inspection agents in a more general form. For example, an experiment's result can also be analyzed by numerical methods such as fittings. In these cases, the *k-agents* framework encourages the users to implement methods that produce reports in texts for the reference of execution agents. The users can use the decorator text_inspection to mark the methods that produce a report for inspecting experiment results. Similarly, the decorator also produces inspection agents that will be called when the experiment is finished.

There might exist many inspection agents in an experiment class, and the reports they produce may be too long for the execution agent to use directly. The class variable _experiment_result_analysis provides an interface to inject the knowledge of how to summarize the reports from the inspection agents. Execution agents use this knowledge to summarize the reports and decide the next stage to transition to.

## 1.2 Representing knowledge of experiment procedures

The knowledge of a laboratory can also exist in experiment procedures, which describe how to decompose an instruction into a procedure that may contain many dependent instructions. To help our agents extract this knowledge more efficiently, we propose the following structured format for them.

**An example of structured experiment procedure**

```
# Complete Calibrating Single Qubit `dut`

## Background

The process of recalibrating a single qubit is crucial to maintaining optimal quantum computation
     performance. This recalibration process involves three key steps: frequency calibration, amplitude
     calibration, and DRAG parameter calibration. Each step is aimed at refining different aspects of the
     qubit's operational parameters to ensure accurate and reliable qubit control. The sequence of these
     steps is critical, as each builds on the stability achieved in the previous step.

## Steps

- Do frequency Calibration on `dut`
- Do amplitude Calibration on `dut`
- Do DRAG Calibration on `dut`

## Results

- Whether the experiments are successful
```

In our format, each experiment procedure is organized in the Markdown format. Each procedure starts from a first-level header, which starts with # and the title of the experiment. Then, our format accepts three sub-sections: Background, Steps and Results, in which the Steps section is necessary. Each sub-section should start from a second-level header as shown in the example.

The Background section is provided to place important background knowledge of the procedure. It can be included in the prompts of the agents to clarify the usage of the procedure.

A procedure translation agent who carries the above information will try to match instructions that are similar to the title of the procedure and modify the procedure in the Steps section to implement the incoming instruction.

Finally, knowledge in the Results section is used to summarize the results of the execution of the procedure and generate a report for the reference of execution agents.

# 2 Supplemental Note 2: Details on implementing agents

We discuss the details of the implementation of the agents in the *k-agents* framework. We especially select important prompts that are used to implement the key functionalities of the agents. We omit the inspection agents as their structure is relatively simple, as we have discussed in previous sections.

## 2.1 Code translation agent

When constructing a code translation agent from an experiment class, a few instructions that match the class will be generated with the following prompt.

**Prompt for generating possible instructions**

```
You are trying to produce imperative sentences that would invoke the execution of action ‘{exp_name}‘
     based on its documentation.
<docstring>
{doc_string}
</docstring>
<example>
Here are a few of examples of imperative sentences:
- Run the calibration experiment with duts=‘duts‘ and start=0.0
- Carry out a calibration on ‘duts‘
- Please execute the Ramsey experiment
- Do the Drag experiment.
</example>
<instruction>
You should output a JSON dict. The keys should be string of indices of the sentences and the values should
      be the sentences.
Each sentence should be complete and independent. Name of the experiment should be transformed to natural
     language and be mentioned.
The sentences should be imperative and should be based on the documentation.
You should output 4 sentences.
</instruction>
```

The instructions will be converted to a list of feature vectors (embeddings) that represent their semantic meaning using an embedding model. These vectors will be used to calculate the matching score of the experiment given a set of instructions to be translated. The score is calculated as the maximum value of the inner product between the embeddings of the instructions to be translated and the generated instructions.

After being activated, the code translation agent will try to generate candidate code that implements the instruction. The prompt for generation is listed below.

**Prompt for generating candidate code**

```
You are trying to call an experiment to fill the code_to_complete in Python. The description of the task
      is written in the slot.
<experiment>
{self.get_exp_description()}
</experiment>
<code_to_complete>
# [slot: {instruction}]
</code_to_complete>
<available_variables>
{available_variables}
```

```
</available_variables>
<requirements>
You should output a JSON dict. The keys should be
- "experiment_name_in_slot" (string): The name of the experiment extracted from the slot.
- "analysis" : The brief analysis of the relation between the experiment and the code_to_complete. You
    should notice that the code_to_complete might be irrelevant to the experiment. You should be careful
    not to assume additional information. The experiment should considered irrelevant if it contains
    extra keywords or irrelevant information.
- "applicable": A boolean whether the experiment you hold is suitable for implementing the task.
- "code": A code snippet that is helpful for filling the slot. The last line of the snippet must be in the
     format: ‘experiment_<name> = {self.exp_cls.__name__}(argument1,argument2, ...)‘. No import
    statements are needed.
- "explanation": A detailed explanation of what the code snippet does based solely on the documentation.
- "suitable": A boolean whether the code snippet matches the task based on the documentation.
</requirements>
```

## 2.2 Procedure translation agent

When initializing procedure translation agents, similar to code translation agents, a few variants of the procedure's title will be generated for their activation.

When activated, a procedure translation agent first checks whether the given instruction to translate matches the procedure title it holds. Then, the agent tries to rewrite the given instruction into a format that is closer to the procedure's title it knows. In the meantime, as many experiment procedures require inputs, the agent also generates a map that maps the available variables in the context to the required inputs of the procedure.

**Prompt for rewriting instructions based on knowledge of an experiment procedure**

```
You are trying to rewrite the following instruction based on your knowledge to suit more general
    parameters.
<input_instruction>
{instruction}
</input_instruction>
The following variables names are defined in the context and you can directly use the variables names.
<available_variables>
{available_variables}
</available_variables>
By your knowledge, there is an existing instruction that is available
<knowledge>
<instruction>
{procedure_title}
</instruction>
</knowledge>
<requirements>
You are required to output a JSON dict with the following keys
- "parameter_specification" (string): The specification of the parameters of the input_instruction
- "analysis" (string): An analysis of the relation between the input_instruction and your knowledge. You
    should notice that your knowledge is likely to be improper if the experiment name contains different
    keywords. However, the parameter_specification of the experiment can be different.
- "proper" (bool): Whether the input_instruction can be regarded as a proper instance of the experiment in
     your knowledge.
- "rewritten_instruction" (string): The input_instruction rewritten in a way based on the instruction in
    your knowledge. You should not change the parameter specification of the input_instruction.
- "parameter_mapping" (dict): A mapping from the parameters in the input_instruction to the parameters in
    the rewrote_instruction. The keys should be the parameters in the rewrote_instruction and the values
    should be the parameters in the input_instruction or the value from the description.
- "annotation" (string): A concise annotation that describe how you are confident that the
    rewritten_instruction is correct.
</requirements>
```

After the rewriting, the procedure translation agent wraps the rewritten instruction into a new experiment that runs the instruction with a new execution agent. The code that calls the new experiment is sent to the execution agent as a code candidate.

## 2.3 Execution agent

Given an experiment procedure, an execution agent will decompose it into stages. Specifically, the decomposition is implemented by a pipeline of LLM calls. The core step in the pipeline is to extract the stages with all the other information dropped.

**Prompt for stage extraction**

```
<experiment_description>
{description}
</experiment_description>
<objective>
Your objective is to decompose the experiment description into standalone instruction.
Each instruction should include an experiment.
The instructions should make a minimal modification to the original description.
You should not make any inferences or interpret the description.
You are encouraged to copy the description as is.
You should output as few instructions as possible. You must not expand the instructions.
The instructions must not contain any information about what to do next after the instruction, such as a
     change of parameter and go to fail.
</requirements>
<example>
For example, if a piece of description is:
"Run experiment A with frequency=10. If failed, retry 3 times."
You should change it into:
"Run experiment A with frequency=10."
<output_format>
You are required to output a JSON dict with a single key "instructions", which contains a list of
     instructions. Each instruction should be represented as a string.
</output_format>
```

After the extraction, the generated stages are input to LLM again to generate the corresponding transition rules. We also add additional steps to extract the numbers in the instructions and save them as a key-value map in each stage. This allows the execution agent to change these numbers in a state transition when needed.

## 2.4 Execution agent

After executing the experiment in a stage, the execution agent decides the next stage to transition to based on the summary of the reports from the inspection agents. The prompt for deciding the next stage includes the summary, the information of the current stage, including how many times it has been executed, and transition rules. A maximum retry limit has been included in the prompt to prevent the model from entering an endless loop.

**Prompt for stage transition**

```
You are analyzing experiment result from current stage and use the rule of transition to determine the
     next stage of the experiment.

<current_stage>
{current_stage.label}:{current_stage.description}
The current stage has been executed {current_stage.n_executed} times.
There are {current_stage.n_failed} failed attempts and {current_stage.n_success} successful attempts.
</current_stage>

Here are the results from the experiments. Note that results must be consistent to indicate the validity.
Otherwise they are both invalid.
<experiment_reports>
{result_prompt}
</experiment_reports>

<rule_of_transition>
{rules}
</rule_of_transition>

<requirements>
Return your decision in JSON format With the following keys:
"analysis" (string): an analysis of the results and the rule of transition to determine the next stage.
"next" (string): the name of the next stage.
</requirements>
```

After deciding on the next stage, we also include additional steps to modify the variables in the next stage because the inspection agent might propose modifying the parameters. This step is especially important when an instruction needs to be retried with a different set of parameters.

# 3 Supplemental Note 3: Benchmarking our methods

## 3.1 Benchmarking translation

To test the performance of translating instructions into code, we manually make a test set that consists of 80 pairs of experiments and instructions. This dataset is for benchmarking the performance and it is not required to use our framework in practice. The dataset was not involved in the LLM training. For example, for the NormalisedRabi experiment, which calibrates the driving frequency of a single qubit, we set the following instructions in the test set.

**Examples of the instructions for NormalisedRabi**

```
- Run rabi experiment to calibrate single qubit gate driving amplitudes
- Measure Rabi oscillations to determine single qubit gate driving amplitudes
- Implement Rabi experiment to find pi pulse duration
- Calibrate the driving amplitudes for single qubit gate by Rabi
- Determine single qubit gate parameter using Rabi experiment
- Run Rabi experiment with default parameters on the single qubit ‘dut‘
- Single qubit gate amplitudes estimation using Rabi experiments
- Do Rabi experiment to measure single qubit drive amplitudes
- Run Rabi experiment on single qubit ‘dut‘ with amp=0.3
- Calibrate single qubit drive amplitudes using Rabi experiment’
```

In *k-agents*, the translation is done by calling the related translation agents, and the execution agent picks one of the code candidates from the translation agents. Specifically, this selection is implemented by inputting all the code candidates, together with the instruction to translate, to an LLM for analysis (See Algorithm 1). To implement the standard RAG implementation of this process, after retrieving the related translation agents, we extract the experiment classes from them and use the signature and documentation of these classes to generate a translation of the instruction (See Algorithm 1 for detail). The same process of embedding-based retrieving ensures that the increase in performance is caused by our agent-based reflexion and selection process. Both our approach and the RAG baseline operate by generating a pool of candidates and then selecting only the single best output for evaluation. In our framework, each agent reasons over all candidates, propose and pick the most suitable translation. The RAG method, retrieves the top candidate based on cosine similarity in embedding space. Because both methods ultimately choose exactly one candidate per query, comparing their final translation accuracies reflects how reasoning-based aggregation can improves accuracy.

---

**Algorithm 1:** Translation with standard RAG

1. Set the group of translation agents $\{A\}$, the number $N_k$ for the number of activated agents and the number $N_{\max}$ for the maximal number of activated agents.
2. Input the instruction $I$ to be translated.
3. Calculate the score of each agent in $\{A\}$ by Equation 1.
4. For each agent $A$ whose score is ranked top $N_k$ in $\{A\}$, add the experiment description it carries to $\{C\}$.
5. Input the instruction and the experiment descriptions in $\{C\}$ to LLM and generate the code $C^*$ for $I$.
6. Output $C^*$ as the result of translation.

---

For a fair comparison, we only included code translation agents in the benchmark as the

information as the prompt for code generation with procedures is different from that with experiment classes (See Section 2.2). Finally, we include 17 experiment classes in the benchmark. When triggering the translation agents, we select the 2 agents with the highest score. If there is no available code candidate, we increase the number of agents to trigger by 2. Accordingly, we set 2 as the number of experiment classes retrieved in the RAG method being compared. By limiting each retrieval to just 2 candidates, we minimize the number of items processed per step and show the scalability of our memory architecture

After the instructions are translated into code, we check the correctness of the code by analyzing its abstract syntax tree. A search for a function call to the correct experiment will be carried out. If such a function call is not found, the translation is marked as incorrect. If a function call to the correct experiment exists, the format of the call will be further checked to ensure the call is valid. Translations that pass this check will be marked as correct.

We additionally note that we use the text-embedding-3-large model from OpenAI as the embedding model throughout all the experiments, even when the LLM we use is not from OpenAI. We use the same embedding model to control the variables and compare differences just in the performance of LLMs.

## 3.2 Comparison with long-prompt apporach

Many large language models (LLMs) now support long inputs, enabling them to process entire codebses. However, aside from the significant cost implications, prior studies have shown that providing long inputs can impair LLM performance[1–3]. For instance, tool-use performance may degrade substantially when too many tools are included in the prompt. This implies that systems like ChemCrow[4] and CoScientists[5]—both of which present all the available tools to the model at once-may face scalability issues. To address this, and in addition to the benchmark introduced in the previous section, we conduct another benchmark to compare our agent-based translation method with long prompt-based methods.

In the benchmark, to evaluate the ability of models to handle long inputs, we inserted the descriptions of each of the 17 experiments twice with a random order, resulting in inputs totaling around 11,000 tokens—exceeding the input limits of many LLMs such as GPT-4 (first release) and LLaMA-3-70B. For a fair comparison, we set the number of agents recalled during each translation to nine, meaning that nine translation agents were activated whenever an instruction needed to be translated. The results, shown in Figure S1, indicate that long-context methods can perform well in many cases; for instance, both GPT-4o and Gemini 1.5 Pro achieved high translation accuracy. However, this high performance is not consistent across all models. For example, Claude 3 Opus exhibited a notable decline in instruction-following ability with longer inputs, leading to poor translation results. In contrast, even when only a subset of translation agents is activated, these agents can still outperform their long-context counterparts. Our result shows that activiating more translation agents can provide better accuracy than inputting all the tools to LLMs. A comparison of translation success rates using a long context versus our agent-based approach is shown in Figure S1.

## 3.3 Benchmarking visual inspection

Here, we describe the method for benchmarking the performance of inspection agents to assess their ability to determine the success of an experiment. The agent has three methods for evaluating success: (1) by reading only the fitting report generated by human-written code, (2) by analyzing only the figures generated from the experimental data, or (3) by first analyzing

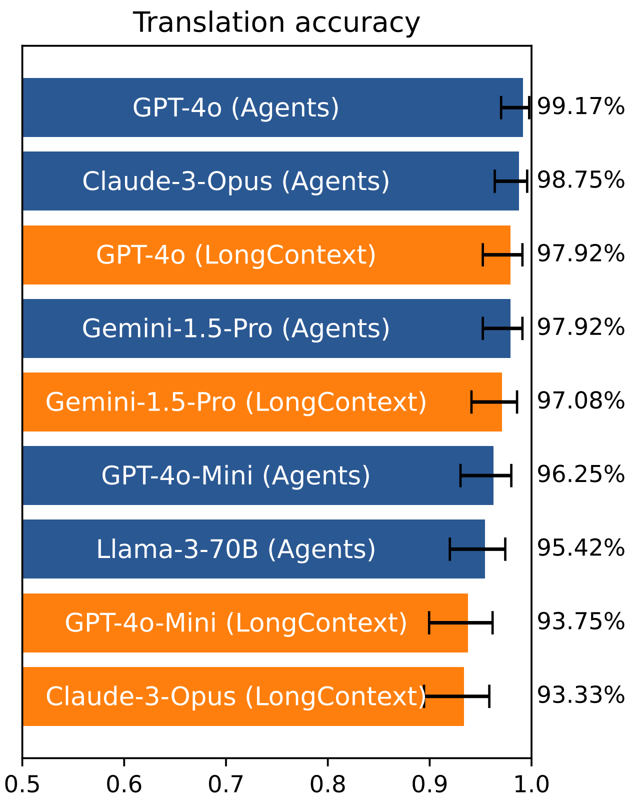


Figure 1: Comparison of translation success rates using a long context versus our agent-based approach. Long context were found to significantly reduce translation accuracy under certain conditions, such as in the case of Claude 3 Opus, highlighting the advantage of our method.

the figures and generating a report, then combining this report with the fitting results to assess success. These methods are referred to as “Fitting”, “Visual”, and “Combined”, respectively.

It is challenging to precisely define success criteria using natural language for the inspection of plots. To address this, we employ a few-shot prompting approach. This involves providing the agent with example images of a successful experiment and a failed experiment, followed by a new image. The agent is asked to determine whether the new image more closely resembles the successful or failed example, with some guidance from natural language descriptions.

The benchmarking dataset consists of four typical experimental results representing common types of plots in quantum computing experiments. To evaluate the agents, we specifically crafted a synthetic dataset where it is difficult to assess success based solely on fitting results—highlighting the need for language model assistance. Each experiment produces 100 successful and 100 failed examples, and the agent is tasked with determining whether each experiment is successful or not. Detailed prompts and reference images are provided below.

**Rabi experiment** The rabi experiment represents a set of examples where we expect to observe a set of oscillations. To observe these oscillations, we do a Fourier transform of the input data and plot its spectrum for the LLMs to determine its correctness. Benchmark one sample of this experiment costs on average 6,056 input tokens and 1,220 output tokens.

**Examples of the instructions for** NormalisedRabi

```
Analyze this quantum mechanics Rabi oscillation experiment plot in the Fourier frequency domain. A
     successful experiment should have a significant peak in the figure.

For example , the following Image is a successful Rabi oscillation experiment plot :
```

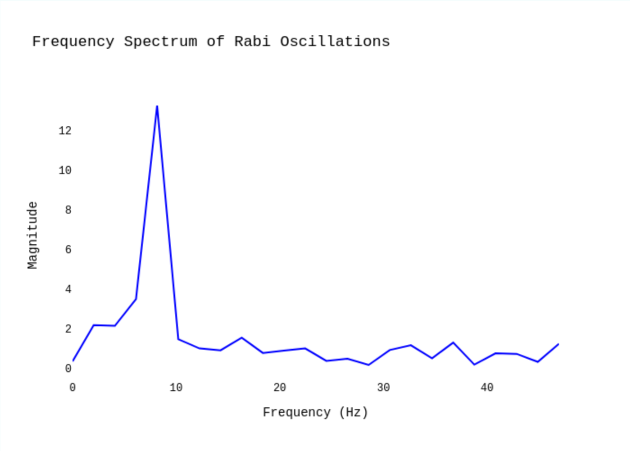


```
the following Image is a failure case for the Rabi experiment :
```

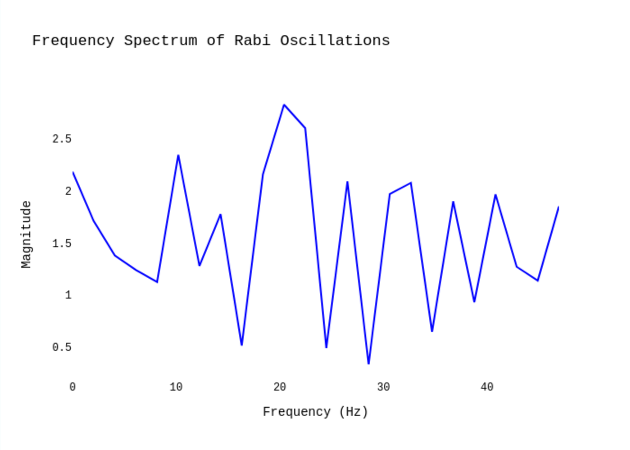

**Resonator spectroscopy** The resonator spectroscopy experiment involves looking for a resonator feature in a 1D space. The failure example observes noise and cannot find a clear resonator. Benchmark one sample of this experiment costs on average 6,886 input tokens and 1,365 output tokens.

**Examples of the instructions for** ResonatorSpectroscopy

```
Analyze the resonator spectroscopy magnitude plot to determine if it exhibits characteristics indicative
    of a resonator.
Specifically, look for a sharp dip or peak in the plot, which would signal the presence of a resonator.
The rest of the plot should remain relatively stable, without significant fluctuations.
Provide a detailed analysis of the magnitude and frequency data. Identifying a resonator indicates a
    successful experiment.

For example , the following Image is a successful experiment plot :
```

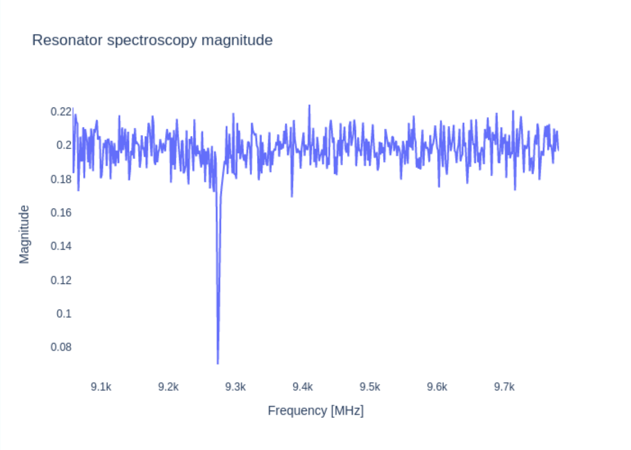


```
the following Image is a failure case for the experiment :
```

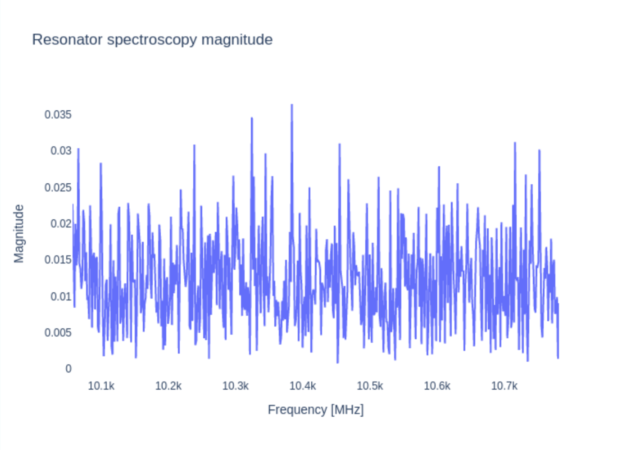


**Qubit Readout with Gaussian Mixture model** The experiment visualizes the integrated readout signal on the IQ plane. Typically, the effectiveness of a Gaussian mixture model can be assessed using a spherical Gaussian mixture model (where the covariance matrix is diagonal) by examining the signal-to-noise ratio (SNR), which is the distance between the Gaussian centers divided by the average standard deviation of the two distributions. However, this approach becomes less effective when there are more than two clusters in the response signal, which can occur if the resonator is overdriven or the driving frequency excites higher energy states. In such cases, using the SNR as a metric for model accuracy is not straightforward. In this synthetic dataset, we introduce scenarios where higher excited states may exhibit small leakage or significant population. Describing such complex criteria in natural language also poses challenges.

Benchmark one sample of this experiment costs on average 7,840 input tokens and 1,857 output tokens.

**Examples of the instructions for** Gaussian Mixture Model readout

```
Analyze a plot of collected signal data to determine experiment success:
1. Identify clusters: The signal represents hidden system states, with each state generating a 2D Gaussian
     distribution (spherical blobs).
2. Count and evaluate distributions:
    - Treat partially overlapped clusters with two visible density centers as separate distributions.
    - Consider elliptical distributions with only one visible density center as a single distribution.
    - Compare densities of observed distributions.
    - If three or more distributions are present, but only two have major density, consider only the two
        high-density distributions and ignore the low-density ones.
3. Experiment outcome:
    - Success: Exactly two major distributions observed (after accounting for density).
     - Failure: Any other outcome (e.g., one distribution, or more than two major distributions).
For example , the following Image is a successful experiment plot :
```

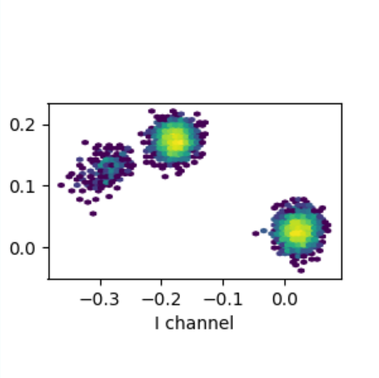


```
the following Image is a failure case for the experiment :
```

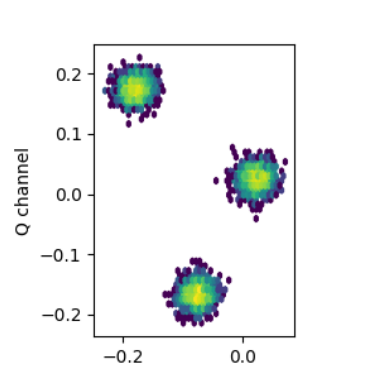


**DRAG experiment** The goal of the DRAG experiment calibration is to identify the intersection point of two lines. We synthesize a dataset where the fitting process will always predict a crossing point between the blue and red lines, though the data may be too noisy to be reliable. The language model is designed to assess whether the input data from the experiment is valid and whether the fitting result can be trusted. Benchmark one sample of this experiment costs on average 7,899 input tokens and 1,980 output tokens.

**Examples of the instructions for** DRAG

```
Analyze the scatter plot with blue and red data points and trend lines:
    1.Compare the slopes of the trend lines.
    2.Assess how well data points fit their trend lines, noting outliers or patterns.
    3.Evaluate data point distribution along the DRAG coefficient axis.
    4.Determine if trend lines accurately represent their datasets.
    5.Compare trends between the two datasets.
    6.Estimate the fitting residuals.
Success criteria:
```

```
    1.Distinct trends for each color
    2.Appropriate line fitting, with the blue and red lines has significant difference in distribution.
    3.Lines intersect near the plot's center region, small shifts away from the center is acceptable.
    4. Residuals are within acceptable range.
If criteria aren't met, mark the experiment as failed and suggest a new range for the sweep.
For example , the following Image is a successful experiment plot :
```

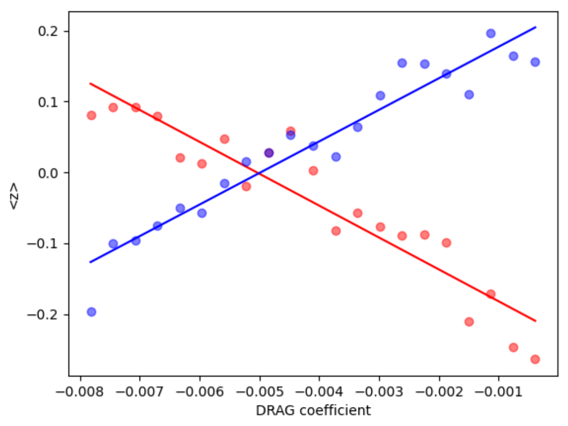


```
the following Image is a failure case for the experiment :
```

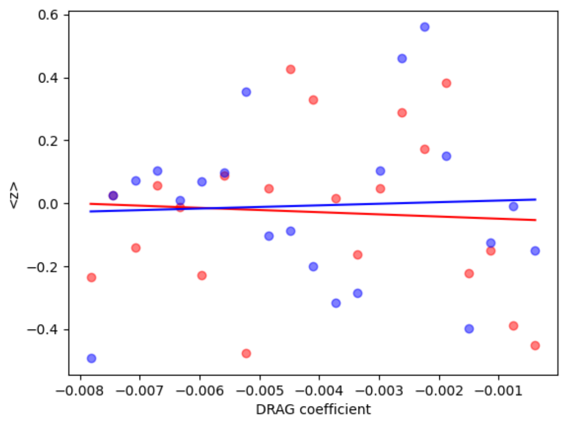


We additionally note that the Llama-3-70B+LLaVA-v1.6-34B setup in the figure uses the Llama 3 70B model[6] when there is no image involved. When inspecting a figure, we adopt an LLaVA 1.6 model[7,8] using the Nous-Hermes-2-Yi-34B model[9] as its base model. As LLaVA 1.6 does not support including multiple images in the conversation, we only demonstrate its performance with the zero-shot prompt for visual inspection.

# 4 Supplemental Note 4: Built-in experiments

Here we detail the implementation of the built-in experiments for single qubit calibration and provide details on how the language model gets prompted. These experiments are standard for superconducting quantum processor calibration, please refer to[10] for more details. For the two-qubit gate related built-in experiments, please refer to appendix 5.

**Ramsey experiment** The Ramsey experiment measures the qubit frequency and coherence time of a qubit by applying two $\pi/2$ pulses separated by a free evolution period, during which the qubit's phase oscillation reflects the offset between the driving frequency and the qubit frequency.

**Ramsey experiment example**

**Execution agent (input)**: Conduct the Ramsey experiment with parameters ”frequency offset=0.1 MHz”, ”stop time=35 microseconds”, ”step size=0.5 microseconds”.

---

**Code translation agent (output)**:

```
experiment_ramsey= SimpleRamseyMultilevel(dut=dut, set_offset=0.1, stop=35, step=0.5)
```

---

**Experiment plot (input)**:

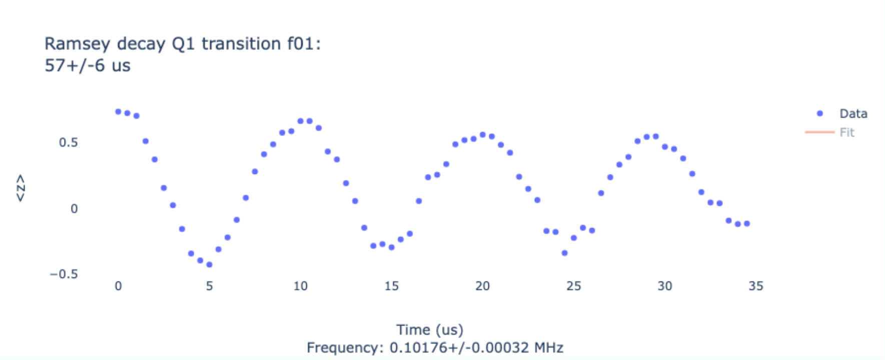


**Fitting (input)**: The Ramsey experiment for qubit Q1 has been analyzed. The expected offset was set to 0.100 MHz, and the measured oscillation is 0.102+/-0.000 MHz. Oscillation amplitude is 0.599+/-0.019. The number of oscillations is 3.562+/-0.011.

---

**Visual inspection agent (output)**: The plot shows data from a quantum mechanics experiment involving Ramsey oscillations. The data points exhibit a clear, regular oscillatory pattern, indicating successful oscillations. The fit line closely follows the data points throughout the plot, suggesting a good fit quality. The data points are relatively tightly clustered around the fit line, with some minor dispersion. The amplitude of the oscillations is around 0.8, which is close to the ideal value of 1, and the frequency is approximately 0.10176 MHz, which is within the expected range. Overall, the plot demonstrates the typical characteristics of a successful Ramsey oscillation experiment, with clear oscillations, a good fit, and consistent amplitude and frequency.

**Final analysis (output)**: The Ramsey experiment for qubit Q1 was successful. The plot shows a clear, regular oscillatory pattern with a good fit. The amplitude of the oscillations is 0.599, which is slightly lower than the ideal value of 1 but still within an acceptable range. The frequency of the oscillations is 0.102 MHz, which is very close to the expected offset of 0.100 MHz. The number of oscillations observed is 3.562, which is within the acceptable range (greater than 3 and less than 10).

---

**Execution Agent (output)**: The results from the experiment indicate that the Ramsey experiment was successful. The plot shows a clear oscillatory pattern with a good fit, and the final analysis confirms that the frequency and amplitude are within acceptable ranges. The experiment success flag is also set to True, meaning the experiment was successful. Therefore, based on the rules, the next state should be 'Complete'.

**Rabi experiment** The Rabi experiment involves driving a qubit with an external electromagnetic pulse on resonance to the qubit frequency, to induce transitions between its quantum states. The oscillation speed of the experiment can be used to infer the rough optimal single-qubit gate pulse amplitude.

**Rabi experiment example**

**Execution agent (input)**: Conduct the Rabi experiment to determine the Rabi rate for rough amplitude calibration.

---

**Code translation agent (output)**:

```
rabi_experiment = NormalisedRabi(dut_qubit=dut, amp=0.2, start=0.01, stop=0.3, step=0.002, fit=True,
    collection_name='f01', mprim_index=0, pulse_discretization=True, update=True)
```

---

**Experiment plot (input)**:

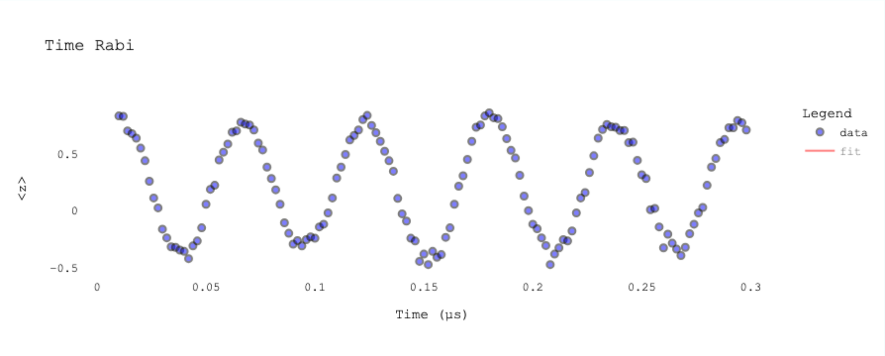


**Fitting (input)**: The fitting result of the Rabi oscillation suggests the amplitude of 0.5893195663534404, the frequency of 17.602131636706986, the phase of 1.5439919053018119. The offset of 0.2087721909927606. The suggested new driving amplitude is 0.19815270673482663.From the fitting results, the plot should exhibit 5.104618174645026 oscillations.

---

**Visual inspection agent (output)**: The plot shows data points that exhibit a clear, regular oscillatory pattern, indicative of Rabi oscillations. The amplitude of the oscillations appears

consistent, with peaks and troughs occurring at regular intervals. The frequency of the oscillations also seems stable throughout the time range. Overall, the plot demonstrates the typical characteristics of successful Rabi oscillation experiments, with well-defined oscillations and consistent amplitude and frequency.
**Final analysis (output)**: The experiment was successful. The plot shows a clear, regular oscillatory pattern with consistent amplitude and frequency, which is characteristic of a successful Rabi oscillation experiment. The fitting results suggest an amplitude of 0.5893, a frequency of 17.6021, and a phase of 1.5440. The plot should exhibit approximately 5.1 oscillations, which is within the acceptable range of 5 oscillations.

---

**Execution Agent (output)**: Transitioning to the next stage Stage2 with the following description: Conduct the Pingpong experiment. The experiment was successful based on the results provided. The plot shows a clear, regular oscillatory pattern with consistent amplitude and frequency, which is characteristic of a successful Rabi oscillation experiment. The fitting results are consistent with the plot, and the final analysis confirms the success of the experiment. Since the experiment was successful, the next stage should be Stage2 (Pingpong Experiment) as per the rules.

**Pingpong experiment** The ping-pong experiment repeatedly applies sequences of quantum gates to estimate the precise amplitude of the single-qubit drive pulse.

**Pingpong experiment example**

**Execution agent (input)**: Conduct the Pingpong experiment.

---

**Code translation agent (output)**:

```
experiment_pingpong = AmpPingpongCalibrationSingleQubitMultilevel(dut=dut, iteration=9, points=10,
     mprim_index=0, repeated_gate='X', flip_other=False, ai_inspection=True)
```

---

**Experiment plot (input)**:

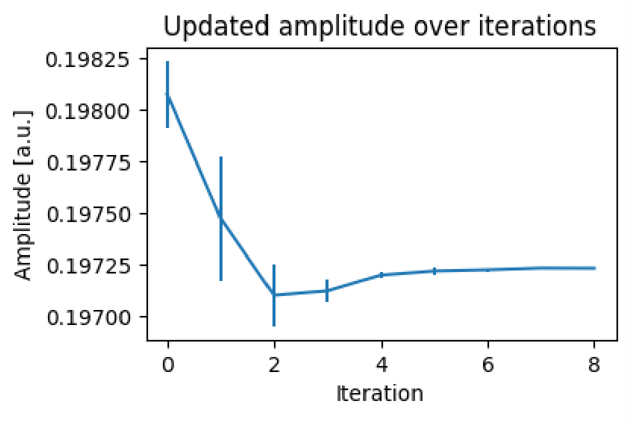

---

**Visual inspection agent (output)**: The amplitude plot shows a decreasing trend initially, followed by a stabilization around the 5th iteration. This indicates that the amplitude converges through the iterations, as the values become relatively constant after the initial decrease.

**Final analysis (output)**: The amplitude plot shows a decreasing trend initially, followed by stabilization around the 5th iteration, indicating that the amplitude converges through the iterations. This suggests that the experiment was successful in achieving fine calibration of the single qubit pulses.

---

**Execution Agent (output)**: The results from both the plot and Final analysis indicate that the amplitude converges and stabilizes around the 5th iteration, which suggests that the experiment was successful in achieving fine calibration of the single qubit pulses. Additionally, the 'Experiment success' result is marked as True, confirming that the experiment was successful. Therefore, based on the rules, the next stage should be 'Complete' as the experiment has been successful.

**DRAG calibration experiment** The DRAG (Derivative Removal by Adiabatic Gate) calibration experiment we used in this experiment reduces phase errors during gate operations by sweeping the DRAG parameter of the pulse.

**DRAG experiment example**

**Execution agent (input)**: Conduct the DRAG calibration on the single qubit gate.

---

**Code translation agent (output)**:

```
experiment_drag = DragCalibrationSingleQubitMultilevel(dut=dut, collection_name='f01', mprim_index=0,
    initial_lpb=None, N=1, num=21)
```

---

**Experiment plot (input)**:

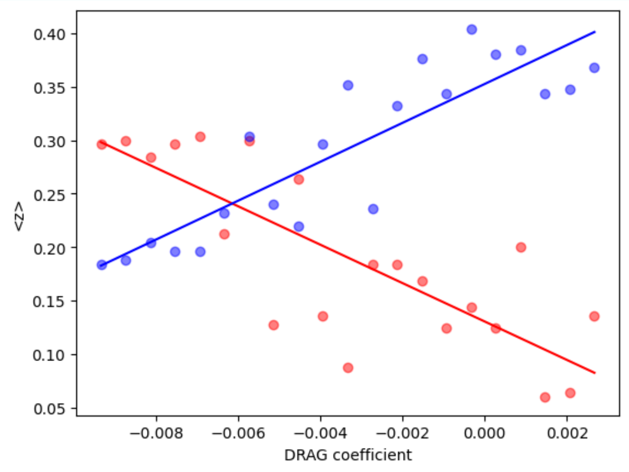


**Fitting (input)**: Sweep start: -0.009333333333333334 Sweep stop: 0.0026666666666666666 The fitting results are as follows: The estimated optimal DRAG coefficient is -0.0061392677674084704 The estimated optimal DRAG coefficient falls within the central half of the sweep.Residual average for Xp: 0.0023451666048237476, Residual average for Xm: 0.0012794699649556793

---

**Visual inspection agent (output)**: 1. The slope of the blue trend line is positive, indicating an increasing trend, while the slope of the red trend line is negative, indicating a decreasing trend. 2. The data points for both colors generally fit their respective trend lines, though there are a few outliers, particularly among the red data points. 3. The data points are distributed along the DRAG coefficient axis, with blue points more concentrated on the right side and red points more concentrated on the left side. 4. The trend lines appear to accurately represent their datasets, capturing the overall direction of the data points. 5. The trends between the two datasets are distinct, with one increasing and the other decreasing. 6. The fitting residuals seem to be within an acceptable range, though there are some deviations, particularly among the red data points.

**Final analysis (output)**: The experiment shows distinct trends between the two datasets, with the blue trend line increasing and the red trend line decreasing. The fitting residuals are within an acceptable range, although there are some deviations, particularly among the red data points. The estimated optimal DRAG coefficient is -0.0061, which falls within the central half of the sweep range, indicating that the sweep was appropriately centered. Overall, the experiment meets the success criteria.

---

**Execution Agent (output)**: The experiment has been successful based on the results provided. The final analysis confirms that the experiment meets the success criteria, with the estimated optimal DRAG coefficient falling within the central half of the sweep range and the residuals being within an acceptable range. Additionally, the 'Experiment success' result is marked as 'True', indicating no need for further retries or adjustments.

**Randomized benchmarking experiment** The randomized benchmarking experiment evaluates the error rates of quantum gates by applying a sequence of random gate operations followed by their inverse, measuring how well the system returns to its initial state[11–13].

**Randomized benchmarking example**

**Execution agent (input)**: Measure the single qubit gate fidelity using randomized benchmarking.

---

**Code translation agent (output)**:

```
experiment_rb = SingleQubitRandomizedBenchmarking(dut=dut, collection_name='f01', seq_length=1024, kinds
     =10, cliff_set='XY', pi_half_only=False, mprim_index=0, seed=42)
```

---

**Experiment plot (input)**:

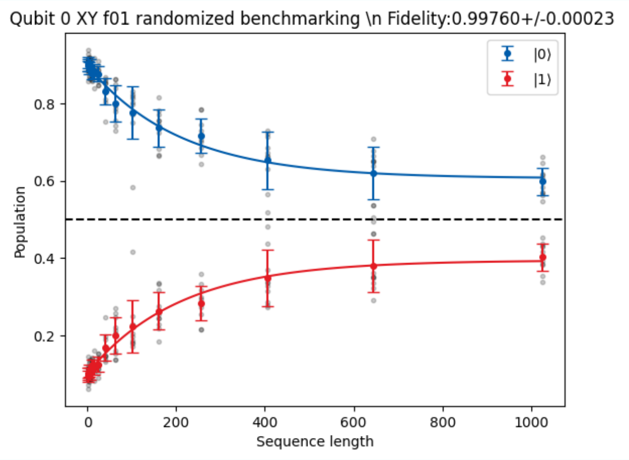


**Fitting (input)**: The randomized benchmarking experiment suggest the infidelity per clifford is 0.00240+/-0.00023 and the infidelity per gate is 0.00131+/-0.00013.

---

**Visual inspection agent (output)**: The plot shows the population of the $|0\rangle$ state (blue) and $|1\rangle$ state (red) as a function of sequence length in a randomized benchmarking experiment. The $|0\rangle$ state exhibits a clear exponential decay, indicating that the experiment is successful. The decay rate appears to be neither too fast nor too slow, suggesting that the sequence length is appropriate.
**Final analysis (output)**: The experiment shows a clear exponential decay in the population of the $|0\rangle$ state, indicating that the experiment is successful. The infidelity per Clifford and per gate are within reasonable physical limits, suggesting that the experiment was conducted properly.

---

**Execution Agent (output)**: The results from the experiment indicate a successful measurement. The plot shows a clear exponential decay in the population of the $|0\rangle$ state, which is consistent with the expected behavior in a randomized benchmarking experiment. The infidelity per Clifford and per gate are within reasonable physical limits, and there are no suggested parameter updates. Additionally, the final analysis confirms that the experiment was successful, and the 'Experiment success' flag is set to True. Therefore, the fidelity measurement is successful, and the next stage should be 'Complete'.

# 5 Supplemental Note 5: Two-qubit siZZle gate

The siZZle gate (Stark-induced ZZ by level excursions) is one method to implement two-qubit interactions on fixed-frequency transmon qubits[14–17]. The siZZle gate off-resonantly drives two qubits, inducing a shift in the energy levels of the two-qubit system, which modifies the ZZ interaction. An approximate formula for the ZZ interaction rate, denoted as $\tilde{\nu}_{ZZ}$, is[16]:

$$\tilde{\nu}_{ZZ} = \nu_{ZZ,s} + \frac{2J\alpha_0\alpha_1\Omega_0\Omega_1\cos(\phi_0 - \phi_1)}{\Delta_{0,d}\Delta_{1,d}(\Delta_{0,d} + \alpha_0)(\Delta_{1,d} + \alpha_1)} \tag{1}$$

where $\nu_{ZZ,s}$ is the static ZZ interaction rate without any applied drives. $\alpha_0$ and $\alpha_1$ represent the anharmonicity of the two qubits. $\Delta_{0,d}$ and $\Delta_{1,d}$ are the detunings of the driving frequencies relative to the transition frequencies of the two qubits. $\phi_0 - \phi_1$ is the relative phase between the two drives applied to the qubits. $J$ denotes the capacitive coupling strength between the qubits, while $\Omega_0$ and $\Omega_1$ are the driving amplitudes of the pulses applied to each qubit.

To calibrate a siZZle gate, the objective is to find the optimal method to drive the two qubits such that the accumulation of the $ZZ$ interaction over time is precisely $\pi/4$. This involves adjusting the driving frequency, which determines $\Delta_{0,d}$ and $\Delta_{1,d}$, the driving amplitude (which sets $\Omega_0$ and $\Omega_1$), and the driving time. Since the ZZ interaction is proportional to $\Omega_0\Omega_1$, we typically choose $\Omega_0 = \Omega_1$ in experiments to maximize the ZZ interaction. However, this does not imply that the amplitudes are exactly the same, as variations in cable attenuations and microwave component performance can cause discrepancies. Therefore, to reproduce the above behavior, we request the agent modify the drive amplitude of the first qubit, while our code infers the amplitude for the second qubit based on previous calibration results of single-qubit gates. In the formula, the relative phase of the two drives, $\phi_0 - \phi_1$, is also significant. Due to the experimental setup, the phase reaching the qubits may differ from the phase difference sent by the electronics. In our experiment, we found that our phase was close to the optimal value, so we did not optimize this parameter.

The pulse sequence for implementing a siZZle gate is shown in Figure S2(a). Typically, in the manual calibration approach, a human scientist selects a pair of driving frequency and amplitude of the Stark drive and then observes the stability of the quantum system. The stability is observed by implementing the Hamiltoniain tomography experiment followed by repeated gate tomography experiment, shown in Figure S2(b) and (c), respectively. If the chosen parameters are not optimal, the oscillation of the expectation value of the quantum state tends to be chaotic and noisy. The scientist then reviews the experimental results and, based on empirical knowledge, suggests a new set of parameters to test. This iterative process is often the most time-consuming aspect of tuning a two-qubit gate. Once a stable and clear result is observed—indicating that the quantum system's dynamics are behaving as expected, an automated code can be employed

to determine the necessary time duration, which is the final parameter required to execute the gate.

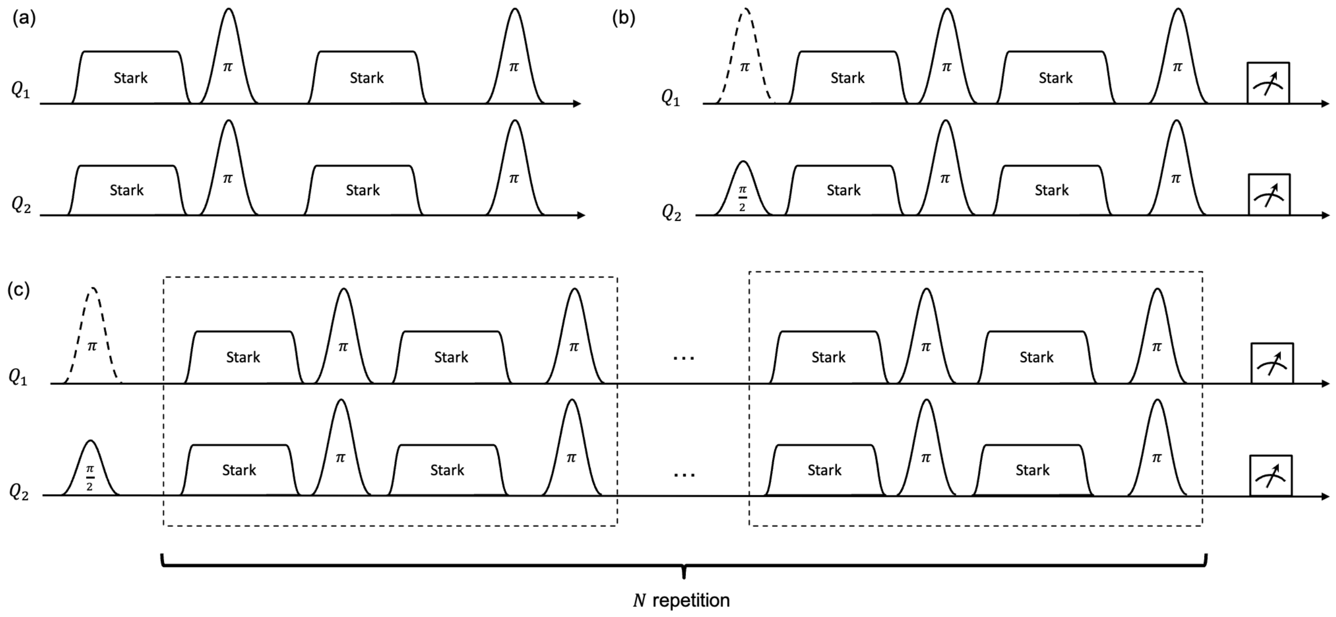


Figure 2: Pulse sequences for calibrating the siZZle gate. (a) The pulse sequence used to implement a single siZZle gate consists of two off-resonance drives to induce a Stark shift (the Stark drive), followed by single-qubit $\pi$ pulses inserted between and at the end of the drive. These $\pi$ pulses are used to cancel the single qubit phase accumulated on individual qubits. (b) The experimental pulse sequence for Hamiltonian tomography to measure the ZZ interaction strength. In this experiment, the pulse width is varied to observe the oscillatory behavior in the phase of the second qubit, which allows inference of the ZZ interaction strength. The dashed line on the pulse for $Q_1$ indicates that the experiment is performed both with and without preparing $Q_1$ in the excited state. (c) Repeated gate tomography experiment. Unlike Hamiltonian tomography, this experiment uses discrete pulses rather than continuously sweeping the width of the Stark pulse. This approach offers more precise calibration of the accumulated ZZ interaction over one repetition block, which can then be used to implement an entangling gate.

To provide the human evaluator with an intuitive understanding of the agent's exploration behavior, we perform a grid scan over the domain of frequency and amplitude, gathering information related to the ZZ interaction strength. While the ideal approach would be directly measuring the interaction strength at each point, this is impractical and too time-consuming. Instead, we employ the pulse sequence shown in Figure S2(b), where the duration of the Stark drive pulse is fixed at $1\mu s$, and we measure the difference in the expectation value when $Q_1$ is prepared to the ground and excited state. The entire experiment takes approximately 12 hours to generate the background figure.

An example of the search iteration on the real hardware is shown as follows. The inspection agent only inspects the control qubit state plot and the Fourier transform of the phase oscillation. The other plots are for humans to evaluate the experiment result.

**siZZle gate parameter search**

**Parameter agent**: Since no experiments have been run yet, we should start by trying a frequency between the two qubits, which is around 4858 MHz. We can start with

a gentle amplitude of 0.2 for the control qubit, which is close to its single qubit gate amplitude. The other parameters are set based on the given rules. params: {'frequency': 4858.0, 'amp_control': 0.2, 'amp_target': 0.0, 'rise': 0.015, 'width': 0.1, 'phase_diff': 0.0, 'zz_interaction_positive': True}

**Translation agent**:

```
experiment_conditional_stark_shift_continuous = ConditionalStarkShiftContinuous(qubits=duts, frequency
     =4858.0, amp_control=0.2, amp_target=0.09943319110052375, rise=0.015, start=0, stop=20,
     sweep_points=40, phase_diff=0.0, echo=True)
```

**Experiment plots**:

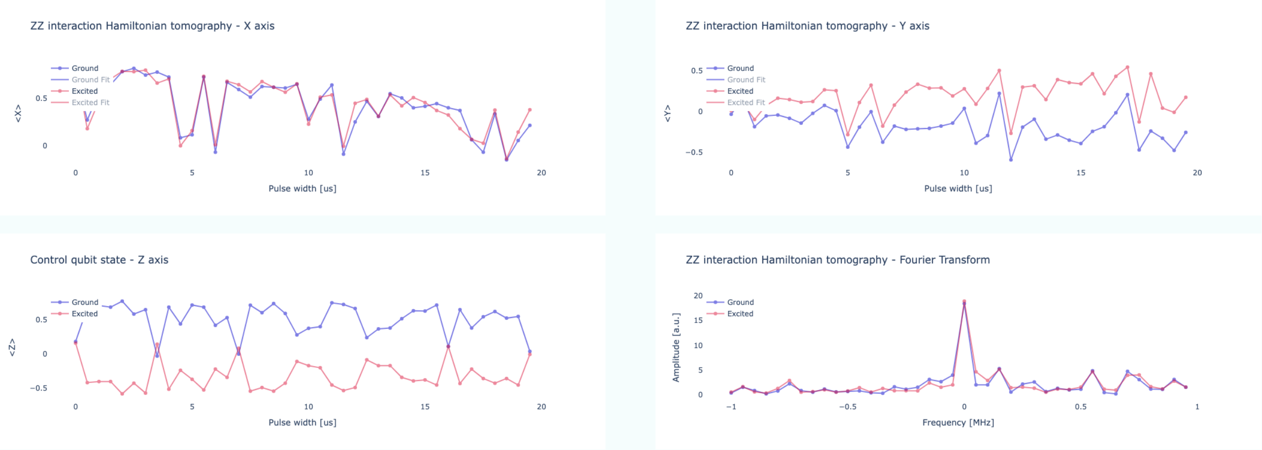


**Inspection agent**: The experiment failed due to the Fourier transform plot showing no clear symmetric peaks around the center frequency for the ground and excited states, indicating unsuccessful measurement of IZ and ZZ interactions.

---

**Parameter agent**: The previous experiment failed because the frequency was too close to the qubits' frequencies. We should try a frequency further away from the qubits. Let's start with a frequency below the lowest qubit frequency and a gentle control amplitude.

```
params: {'frequency': 4800.0, 'amp_control': 0.2, 'amp_target': 0.09776666514167257, 'rise': 0.015, 'width
     ': 1.1730133547479822, 'phase_diff': 0.0, 'zz_interaction_positive': True}
```

---

**Translation agent:**:

```
experiment_conditional_stark_shift_continuous = ConditionalStarkShiftContinuous(qubits=duts, frequency
     =4858.0, amp_control=0.2, amp_target=0.09943319110052375, rise=0.015, start=0, stop=20, sweep\
     _points=40, phase_diff=0.0, echo=True)
```

**Experiment plots**:

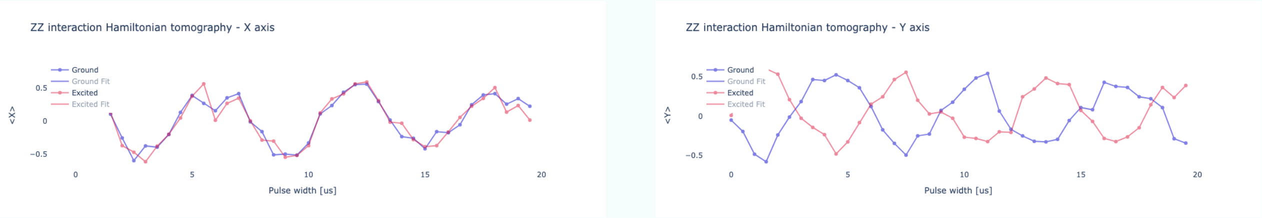

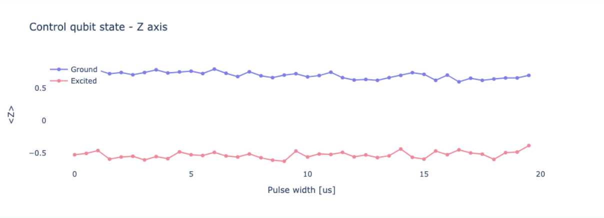


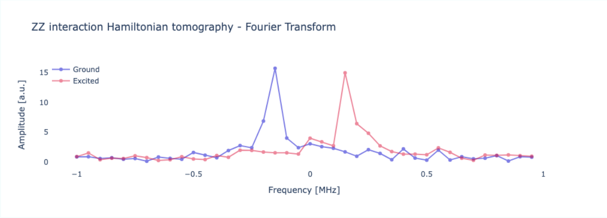


**Inspection agent**: The experiment was successful based on the clear, symmetric peaks in the Fourier plot, stable control qubit expectation value, and reasonable fitting results showing a ZZ interaction strength of 0.16543846027754651 MHz.
**Translation agent**:

```
experiment_result = ConditionalStarkShiftRepeatedGate(duts=duts, frequency=4800.0, amp_control=0.2,
    amp_target=0.09943319110052375, rise=0.015, start_gate_number=0, gate_count=32, width
    =0.3778425958178225, phase_diff=0.0, echo=True)
```

**Experiment plots**:

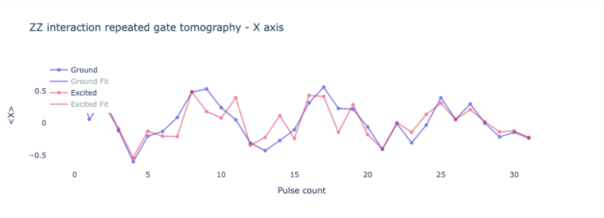


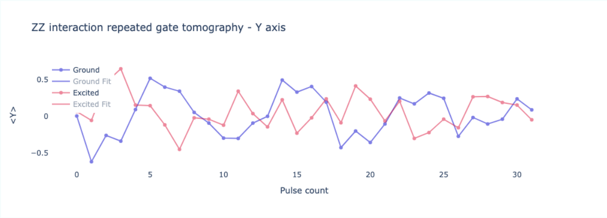


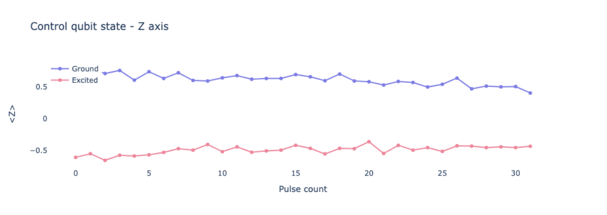


**Inspection agent**: The data inspection AI reports that the Fourier transform plot shows two clear, symmetric peaks for the ground and excited states, and the expectation value of the control qubit along the Z axis remains stable throughout the experiment. The minimum difference between the ground and excited states is greater than 25% of the maximum difference, indicating a successful experiment. The fitting results estimate the ZZ interaction strength to be -0.1170390305626309 MHz, which is a reasonable and physical value. Both the data inspection and fitting results suggest that the experiment was successful.

# 6 Supplemental Note 6: Experimental Hardware and Setup

The quantum device contains 16 transmon qubits in 3D-integrated coaxial circuit quantum electrodynamics (cQED) architecture[18–20]. The details of this device has been reported in[21]. Each qubit consists of a Josephson junction (JJ) and a readout resonator positioned on opposite sides of the chip, enabling precise couplings to off-chip coaxial ports for control and readout. The qubits are arranged in a $4 \times 4$ lattice and are statically coupled through capacitive elements, facilitating interactions characterized by exchange energy rates ($J$) between each pair of qubits. The layout of the chip, depicted in Figure S3(a), illustrates the positioning of the three qubits

used in this work.

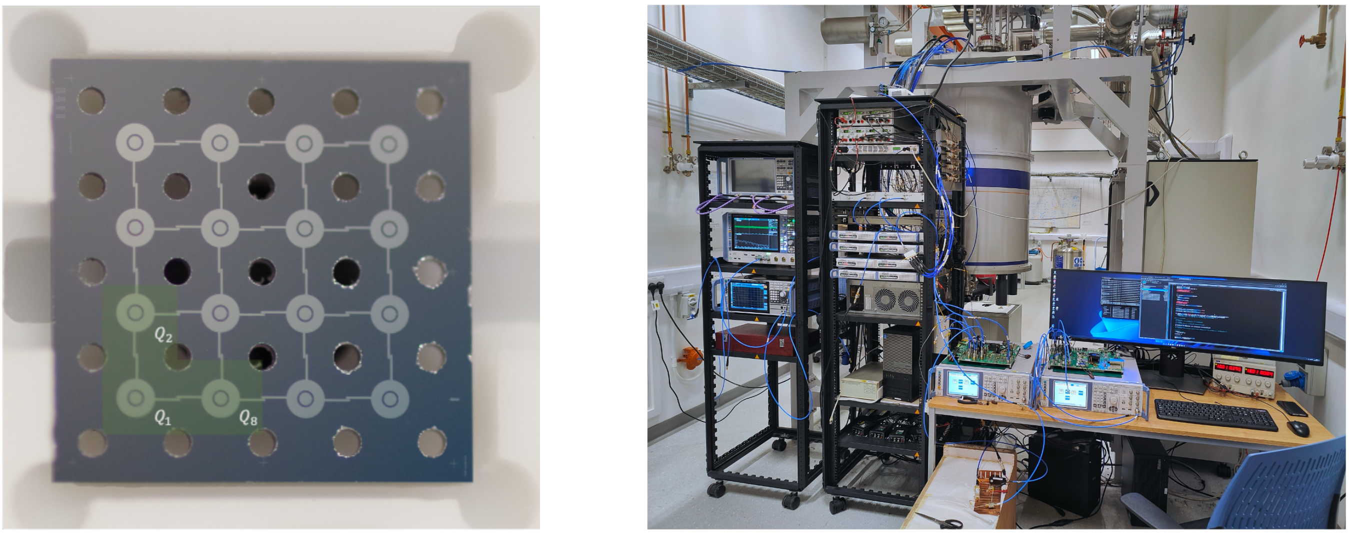


(a) (b)

Figure 3: Hardware setup photos. (a) The photo of the superconducting quantum processor chip used in this experiment. The three qubits involved in this experiment are highlighted in green color. (b) The photo of the hardware setup in the lab.

The basic characterization of the three selected qubits is detailed in Table 1. These qubits operate in the straddling regime, that is, detuning frequencies smaller than the average anharmonicity ($\langle\alpha\rangle = 196.4$ MHz) across the device. This ensures that most qubits remain within a regime where their interactions can be effectively controlled and utilized to drive two-qubit gates[11–13].

Table 1: **Table S1:** Basic characterization of the three qubits used in this work. $Q_{\text{int}}$ is the internal Q factor of the resonator, $\kappa_{\text{ext}}$ is the external decay rate of the resonator, and $\mathcal{F}$ is single-qubit gate fidelity by randomized benchmarking, where all Clifford gates are decomposed into physical X and Y gates.

| Qubit | $\omega_q/2\pi$ GHz | $\omega_r/2\pi$ GHz | $Q_{\text{int}}$ $10^4$ | $\kappa_{\text{ext}}/2\pi$ MHz | $\chi/2\pi$ kHz | $\alpha/2\pi$ MHz | $T_1$ $\mu$s | $T_{2R}$ $\mu$s | $T_{2E}$ $\mu$s | $\mathcal{F}$ % | $J_{Q1-Q2}$ MHz | $J_{Q1-Q8}$ MHz |
|---|---|---|---|---|---|---|---|---|---|---|---|---|
| $Q_1$ | 4.888 | 9.997 | 11.661 | 2.645 | -200 | -197 | 126 (18) | 107 (12) | 124 (23) | 99.993 | 0.760 | - |
| $Q_2$ | 4.795 | 9.386 | 11.785 | 1.337 | -225 | -197 | 89 (13) | 56 (15) | 86 (12) | 99.861 | | 0.702 |
| $Q_8$ | 4.829 | 9.908 | 5.843 | 2.040 | -175 | -197 | 63 (8) | 32 (7) | 71 (8) | 99.944 | - | |

The experimental setup operates at a base temperature of approximately $15$ mK using a $^3$He/$^4$He dilution refrigerator. The control and readout of qubits are facilitated by the wiring configurations shown in Figure S4. For qubit control, microwave pulses are synthesized directly using the QubiC system[22]. See Figure S3(b) for the lab photo. The pulses are carefully shaped for single and two-qubit gates to align at the desired frequencies. For readout, reflected signals from the resonators undergo amplification and down-conversion and are then captured by Analog-to-Digital Converters (ADCs) connected to the FPGA for measurements and further data analysis.

The schematic diagrams of the full experimental setup, including wiring and cryogenic components, are shown in Figure S4.

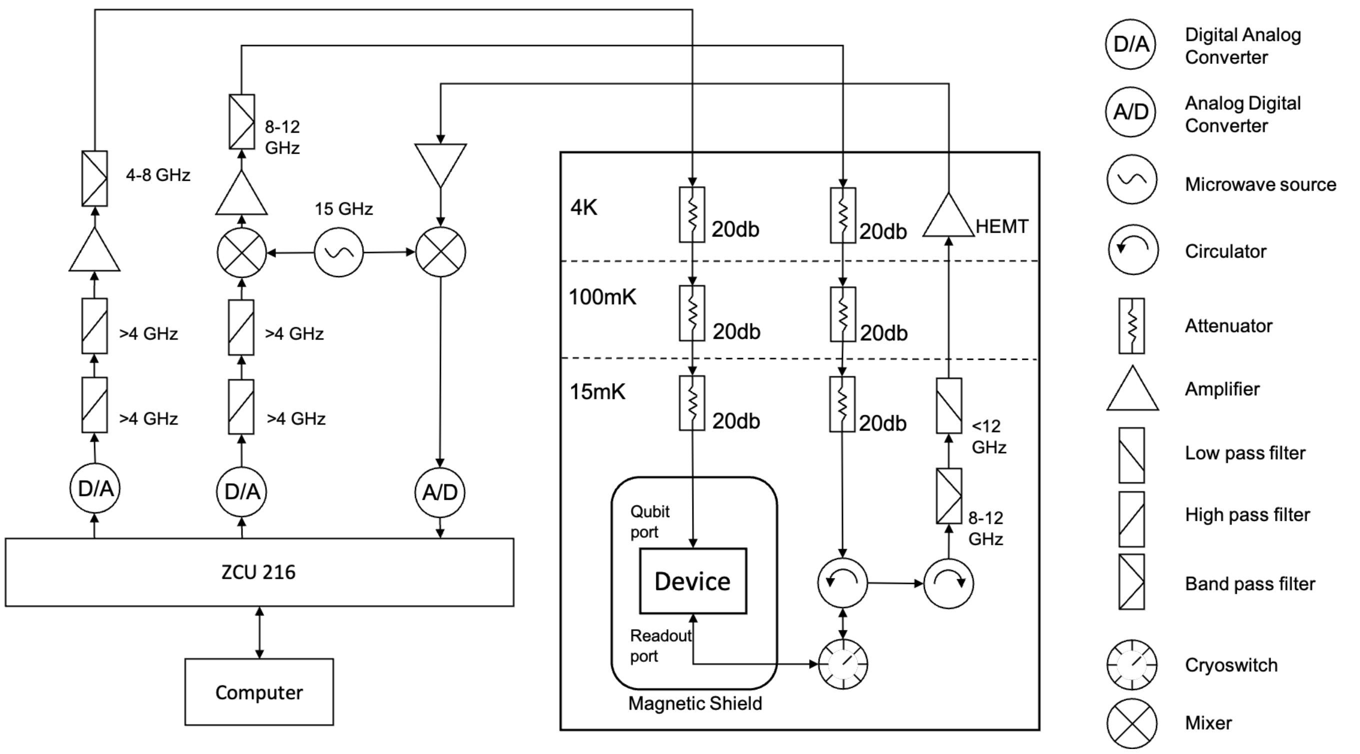


Figure 4: Schematic diagrams of the full experimental setup, including wiring and cryogenic components.

# References


1. Liu, N.F., Lin, K., Hewitt, J., Paranjape, A., Bevilacqua, M., Petroni, F., and Liang, P. (2023). Lost in the middle: How language models use long contexts. arXiv preprint arXiv:2307.03172.

2. Wang, H., Wang, R., Xue, B., Xia, H., Cao, J., Liu, Z., Pan, J.Z., and Wong, K.F. (2024). Appbench: Planning of multiple apis from various apps for complex user instruction. arXiv preprint arXiv:2410.19743.

3. Yao, S., Shinn, N., Razavi, P., and Narasimhan, K. (2024). $\tau$-bench: A benchmark for tool-agent-user interaction in real-world domains. arXiv preprint arXiv:2406.12045.

4. M. Bran, A., Cox, S., Schilter, O., Baldassari, C., White, A.D., and Schwaller, P. (2024). Augmenting large language models with chemistry tools. Nature Machine Intelligence pp. 1–11.

5. Boiko, D.A., MacKnight, R., Kline, B., and Gomes, G. (2023). Autonomous chemical research with large language models. Nature *624*, 570–578.

6. Dubey, A., Jauhri, A., Pandey, A., Kadian, A., Al-Dahle, A., Letman, A., Mathur, A., Schelten, A., Yang, A., Fan, A. et al. (2024). The llama 3 herd of models. arXiv preprint arXiv:2407.21783.

7. Liu, H., Li, C., Wu, Q., and Lee, Y.J. (2024). Visual instruction tuning. Advances in neural information processing systems *36*.

8. Liu, H., Li, C., Li, Y., and Lee, Y.J. (2024). Improved baselines with visual instruction tuning. In Proceedings of the IEEE/CVF Conference on Computer Vision and Pattern Recognition. pp. 26296–26306.

9. Research, N. (2024). Nous hermes 2 - yi-34b. https://huggingface.co/NousResearch/Nous-Hermes-2-Yi-34B. Hugging face.

10. Krantz, P., Kjaergaard, M., Yan, F., Orlando, T.P., Gustavsson, S., and Oliver, W.D. (2019). A quantum engineer's guide to superconducting qubits. Applied Physics Reviews *6*, 021318. URL: https://doi.org/10.1063/1.5089550. doi: 10.1063/1.5089550. arXiv:https://pubs.aip.org/aip/apr/article-pdf/doi/10.1063/1.5089550/16667201/021318_1

11. Emerson, J., Alicki, R., and Życzkowski, K. (2005). Scalable noise estimation with random unitary operators. Journal of Optics B: Quantum and Semiclassical Optics *7*, S347–S352. URL: https://doi.org/10.1088/1464-4266/7/10/021. doi: 10.1088/1464-4266/7/10/021.

12. Dankert, C., Cleve, R., Emerson, J., and Livine, E. (2009). Exact and approximate unitary 2-designs and their application to fidelity estimation. Phys. Rev. A *80*, 012304. URL: https://link.aps.org/doi/10.1103/PhysRevA.80.012304. doi: 10.1103/PhysRevA.80.012304.

13. Magesan, E., Gambetta, J.M., Johnson, B.R., Ryan, C.A., Chow, J.M., Merkel, S.T., da Silva, M.P., Keefe, G.A., Rothwell, M.B., Ohki, T.A., Ketchen, M.B., and Steffen, M. (2012). Efficient measurement of quantum gate error by interleaved randomized benchmarking. Phys. Rev. Lett. *109*, 080505. URL: https://link.aps.org/doi/10.1103/PhysRevLett.109.080505. doi: 10.1103/PhysRevLett.109.080505.

14. Mitchell, B.K., Naik, R.K., Morvan, A., Hashim, A., Kreikebaum, J.M., Marinelli, B., Lavrijsen, W., Nowrouzi, K., Santiago, D.I., and Siddiqi, I. (2021). Hardware-efficient microwave-activated tunable coupling between superconducting qubits. Phys. Rev. Lett. *127*, 200502. URL: https://link.aps.org/doi/10.1103/PhysRevLett.127.200502. doi: 10.1103/PhysRevLett.127.200502.

15. Xiong, H., Ficheux, Q., Somoroff, A., Nguyen, L.B., Dogan, E., Rosenstock, D., Wang, C., Nesterov, K.N., Vavilov, M.G., and Manucharyan, V.E. (2022). Arbitrary controlled-phase gate on fluxonium qubits using differential ac stark shifts. Phys. Rev. Res. *4*, 023040. URL: https://link.aps.org/doi/10.1103/PhysRevResearch.4.023040. doi: 10.1103/PhysRevResearch.4.023040.

16. Wei, K., Magesan, E., Lauer, I., Srinivasan, S., Bogorin, D., Carnevale, S., Keefe, G., Kim, Y., Klaus, D., Landers, W. et al. (2021). Quantum crosstalk cancellation for fast entangling gates and improved multi-qubit performance. arXiv preprint arXiv:2106.00675.

17. Wei, K.X., Magesan, E., Lauer, I., Srinivasan, S., Bogorin, D.F., Carnevale, S., Keefe, G.A., Kim, Y., Klaus, D., Landers, W., Sundaresan, N., Wang, C., Zhang, E.J., Steffen, M., Dial, O.E., McKay, D.C., and Kandala, A. (2022). Hamiltonian engineering with multicolor drives for fast entangling gates and quantum crosstalk cancellation. Phys. Rev. Lett. *129*, 060501. URL: https://link.aps.org/doi/10.1103/PhysRevLett.129.060501. doi: 10.1103/PhysRevLett.129.060501.

18. Spring, P.A., Cao, S., Tsunoda, T., Campanaro, G., Fasciati, S., Wills, J., Bakr, M., Chidambaram, V., Shteynas, B., Carpenter, L., Gow, P., Gates, J., Vlastakis, B., and Leek, P.J.

(2022). High coherence and low cross-talk in a tileable 3d integrated superconducting circuit architecture. Science Advances *8*. URL: https://doi.org/10.1126/sciadv.abl6698. doi: 10.1126/sciadv.abl6698.

19. Rahamim, J., Behrle, T., Peterer, M.J., Patterson, A., Spring, P.A., Tsunoda, T., Manenti, R., Tancredi, G., and Leek, P.J. (2017). Double-sided coaxial circuit qed with out-of-plane wiring. Applied Physics Letters *110*, 222602. URL: https://doi.org/10.1063/1.4984299. doi: 10.1063/1.4984299. arXiv:https://doi.org/10.1063/1.4984299.

20. Alghadeer, M., Fasciati, S.D., Cao, S., Piscitelli, M., Spink, M.C., Hopkinson, D.G., Danaie, M., Speller, S.C., Leek, P.J., and Bakr, M. (2025). Characterization of nanostructural imperfections in superconducting quantum circuits. . arXiv:arXiv:2501.15059.

21. Alghadeer, M., Cao, S., Fasciati, S.D., Piscitelli, M., Gow, P.C., Gates, J.C., Bakr, M., and Leek, P.J. (2025). Low crosstalk in a scalable superconducting quantum lattice. . arXiv:arXiv:2505.22276.

22. Xu, Y., Huang, G., Fruitwala, N., Rajagopala, A., Naik, R.K., Nowrouzi, K., Santiago, D.I., and Siddiqi, I. (2023). Qubic 2.0: An extensible open-source qubit control system capable of mid-circuit measurement and feed-forward. .
